# Porous organic polymers-based fluorescent chemosensors for Fe(III) ions-a functional mimic of siderophores

**DOI:** 10.3389/fchem.2024.1361796

**Published:** 2024-02-15

**Authors:** Binduja Mohan, Ananthu Shanmughan, Alenthwar Vamshi Krishna, Mannanthara Kunhumon Noushija, Deivasigamani Umadevi, Sankarasekaran Shanmugaraju

**Affiliations:** Department of Chemistry, Indian Institute of Technology Palakkad, Palakkad, Kerala, India

**Keywords:** porous organic polymers, luminescence polymer, chemosensors, fluorescence sensing of Fe(III) ions, siderophores

## Abstract

Extended organic polymers such as amorphous Covalent Organic Polymers (COPs) and crystalline Covalent Organic Frameworks (COFs) are emerging functional polymeric materials that have recently been shown promises as luminescent materials for chemosensing applications. A wide variety of luminescence COPs and COFs have been synthesized and successfully used as fluorescence-sensing materials for hazardous environmental pollutants and toxic contaminants. This review exemplifies various COPs and COFs-based fluorescence sensors for selective sensing of Fe(III) ions. The fluorescence sensors are sorted according to their structural features and each section provides a detailed discussion on the synthesis and fluorescence sensing ability of different COPs and COFs towards Fe(III) ions. Also, this review highlights the limitations of the existing organic polymer-based chemosensors and future perspectives on translating COPs and COFs-based fluorescence sensors for the practical detection of Fe(III) ions.

## 1 Introduction

Fe(III) ions are necessary minerals that are widely found in many modern products and are crucial in various physiological processes in biological systems (Abbaspour et al., 2014). These ions play a significant role in a variety of human functions such as the synthesis of hemoglobin, brain and muscle activity, metabolic processes, DNA and RNA transcription, and translation (Eisenstein, 2000; Zheng et al., 2020). Nevertheless, high Fe(III) ion concentration can cause several anomalies and disorders, such as skin conditions, immune system deterioration, and sleeplessness (Ding et al., 2019). Also, neurodegenerative illnesses including Alzheimer’s, Parkinson’s, and Huntington’s diseases are closely linked to the cellular toxicity of Fe(III) ions (Zheng et al., 2013). The permissible concentration of Fe(III) is not more than 0.3 mg/L and any concentration beyond this level is deemed hazardous (Mahmud, et al., 2016). Therefore, it is essential to keep track of the concentration of Fe(III) ions in groundwater. Consequently, a range of sophisticated analytical techniques including inductively coupled plasma atomic emission spectrometry, electrochemical methods, time-of-flight resonance ionization mass spectrometry, and atomic absorption spectroscopy, have so far been employed for the detection of Fe(III) ions (Tangen et al., 2002; Oh et al., 2013; Canfranc et al., 2001). These conventional instrumental techniques, however, are expensive, time-consuming, and often require pre-treatment and trained operators (Canfranc et al., 2001). Therefore, there is a growing need to develop a cost-effective detection method capable of selectively sensing Fe(III) ions even in the presence of other competing metal ions. Recently, the fluorescence-based sensing of Fe(III) ions has become a powerful and alternative sensing method owing to its simplicity, easy visualization, portability, low cost, high sensitivity, and fast response time for detection (Sahoo et al., 2019).

Until recently, a plethora of fluorescence sensors have been developed and successfully used for selective detection and quantification of Fe(III) ions (Segura et al., 2016; Harigae, 2018; Li et al., 2018; Liu et al., 2019). Among the various sensors, small-molecule-based fluorescence sensors have been largely explored for Fe(III) ions detection owing to the advantages including straightforward synthesis, easy purification and good solution processability, better reproducibility, facile structure, and functional tuneability to improve the selectivity of the sensors towards particular analytes (Shanmugaraju et al., 2013). However, the practical applications of discrete small-molecule fluorescence sensors are impaired by their poor sensitivity for detection since they interact/bind stoichiometrically with the target analytes. One facile route to improve the sensitivity of discrete sensors is to link them either covalently or non-covalently to form extended polymeric networks. Due to the long-range exciton communications (called molecular-wire effect), the polymer-based fluorescence sensors are expected to show enhanced sensitivity for analyte detection because one equivalent of analyte can completely quench the fluorescence emission intensity of the sensor (Yang and Swagger, 1998; Gole et al., 2011). One such polymeric material that has been widely investigated for fluorescence-based sensing applications was luminescent COPs and COFs because of their unique properties such as gram-scale synthesis, tunable structure and functional properties, good recyclability, and so on.

Porous organic polymers such as COPs and COFs, in which the organic linkers are connected by strong covalent bonds, are one of the captivating classes of functional materials with interesting characteristics such as low gravimetric density, exceptional thermal and chemical stability, and tunable surface properties (Zhang et al., 2013). COPs and COFs can be easily synthesized by following established organic synthetic methods and their structures and functional properties can be modulated by selecting the appropriate building units, which makes COPs and COFs highly versatile and intriguing functional materials (Kang et al., 2016). In the past decades, the scientific community has shown great research interests in the rational design and targeted synthesis of COPs and COFs with customizable functional properties for their wide range of applications in diverse fields including gas adsorption and separation, drug delivery systems, heterogeneous catalysis, proton conduction, and smart sensing materials. COPs and COFs are a fascinating class of materials with interesting characteristics such as low gravimetric density, exceptional thermal and chemical stability, and tunable surface properties (Fang et al., 2015; Kang et al., 2016; Ma et al., 2016; Han et al., 2017; Medina et al., 2017; Karak et al., 2018; Liu et al., 2019; Wang et al., 2019; Xu et al., 2019; Dai and Liu, 2020; Feng et al., 2020; Giri et al., 2020; Hua et al., 2020; Lee and Cooper, 2020; Wang et al., 2020; Skorjanc et al., 2021; Zhang et al., 2021; Wu et al., 2023; Zhang et al., 2023). The polygonal skeletons of COPs and COFs are completely pre-designable, synthetically controlled, and highly organized throughout the material. It is possible to predetermine their size and shape. This presents an excellent opportunity to develop novel functional polymeric materials. These materials are less soluble in common organic solvents which makes it easier to separate, regenerate, utilize them again, and incorporate them into devices. Owing to their large surface area and pore structure, COPs and COFs materials exhibit enhanced sensing performances compared to small-molecule sensors (Yao et al., 2023). Therefore, luminescence COPs and COFs materials have the potential to revolutionize the field of sensing by offering enhanced sensitivity and selectivity, paving the way for the development of advanced sensing devices for sensing various target analytes. Furthermore, COPs and COFs have the potential to completely transform the sensing industry and open the door for the creation of sophisticated sensing instruments that can detect a wide range of target analytes with excellent selectivity and sensitivity.

In this review article, we provide a comprehensive overview of various luminescent COPs and COFs-based fluorescence chemosensors reported to date for selective sensing of Fe(III) ions. Siderophores are low molecular weight chelators that form selective complexes with Fe(III) ions. On the other hand, COPs and COFs are high molecular-weight polymeric structures. Therefore, we named various COPs and COFs with high affinity for Fe(III) as functional mimics of siderophores. To date, no review articles have been reported exclusively highlighting the fluorescence sensing applications of COPs and COFs-based chemosensors towards Fe(III) ions detection. The different sensors highlighted herein are sorted and listed according to their structural features and each section provides a detailed discussion of synthesis, structures, and fluorescence sensing properties including the sensing mechanism of different COPs and COFs sensors for Fe(III) ions detection.

## 2 Fluorescence sensing mechanisms

**FIGURE 1:**
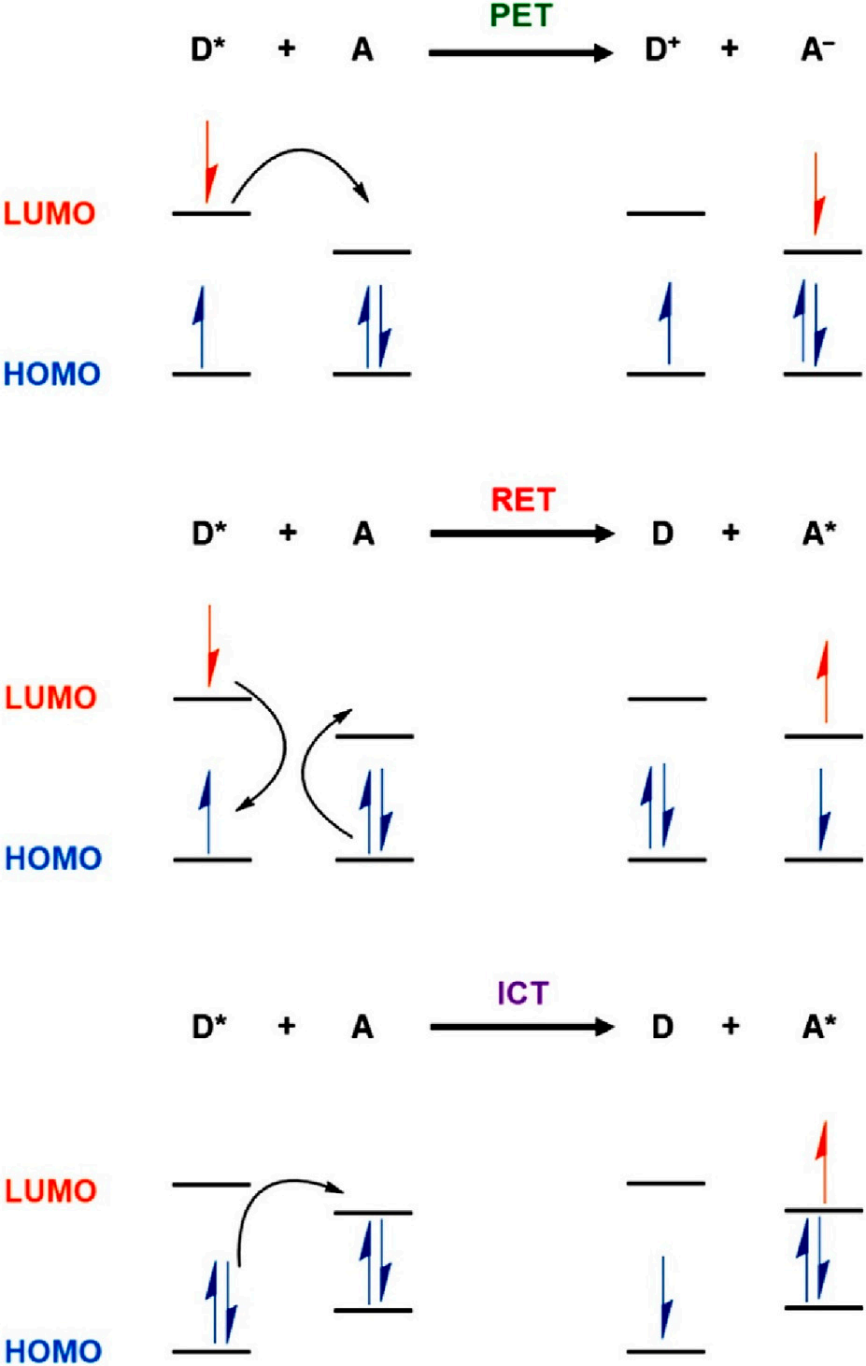
Pictorial representation of commonly proposed fluorescence sensing mechanisms (PET, RET, and ICT) for COP and COF-based sensing of Fe(III) ions [D = Donor (COPs and COFs); A = Acceptor (Fe(III)]. Reprinted with permission from (Skorjanc et al., 2021). Copyright 2021 American Chemical Society.

A typical fluorescence sensor consists of a receptor site for selective binding of analytes and fluorescence indicators for indicating the notable changes upon analytes binding. The receptor and the indicator moieties are either connected directly or through a spacer. The selective binding of targeted analytes at the receptor sites of the photoexcited sensor can induce perturbation in the fluorescence emission intensity either quenched (*turn-off*) or enhanced (*turn-on*) mainly through the excited-state energy or electron transfer. In general, the alteration in fluorescence emission intensity follows two different mechanistic pathways, static and dynamic quenching mechanisms (Shanmugaraju and Mukherjee, 2015). In a static sensing mechanism, the fluorescence sensors interact with the analyte in the ground state via non-fluorescent charge-transfer complex formation and it does not depend on the excited-state fluorescence lifetime of sensor systems. In contrast, in the dynamic sensing mechanism, sensor molecules bind with the analytes in the excited state through molecular collisions and it depends on the rate of molecular collisions and fluorescence lifetime of sensor systems. Therefore, the static and dynamic sensing mechanisms can easily be differentiated by monitoring the changes in the fluorescence lifetime of sensors as the concentrations of targeted analytes increase. Both the sensing mechanisms are often characterized by a linear Stern–Volmer plot which exhibits changes in emission intensity as a function of analyte concentration that allows one to determine the concentration of target analytes. Several types of fluorescence sensing mechanisms like photo-induced electron transfer (PET)—is an excited state electron transfer process in which an excited electron in the donor is transferred to acceptor molecules, resonance-energy transfer (RET)—is the energy transfer process in which electronic energy is transferred from one molecule to another, fluorescence-resonance energy transfer (FRET)—is a process in which energy of an excited state fluorophore is non-radiatively transferred to another fluorophore, intramolecular charge transfer (ICT)—is an excited state process of electron transfer between donor and acceptor moieties, and chelation-induced enhanced fluorescence (CHEF)—is a process in which fluorescence emission intensity is increased multi-fold by ligand chelation effect, have been proposed for COPs and COFs-based sensing of Fe(III) ions. These different fluorescence sensing mechanisms are highlighted in Figure 1 (Skorjanc et al., 2021). In particular, PET is a commonly encountered sensing mechanism for COP and COF sensors for Fe(III) ions detection (Sahoo et al., 2012). In PET, the fluorescence sensors and Fe(III) ions form charge transfer complexes in the excited state and relax back to the ground state by transferring excited state electrons of COPs/COFs to the partially filled d-orbitals of Fe(III) ions resulting in decreases in fluorescence emission intensity (Fu et al., 2018).

## 3 Covalent organic polymers (COPs)-based fluorescent sensors for Fe(III) ions

**FIGURE 2:**
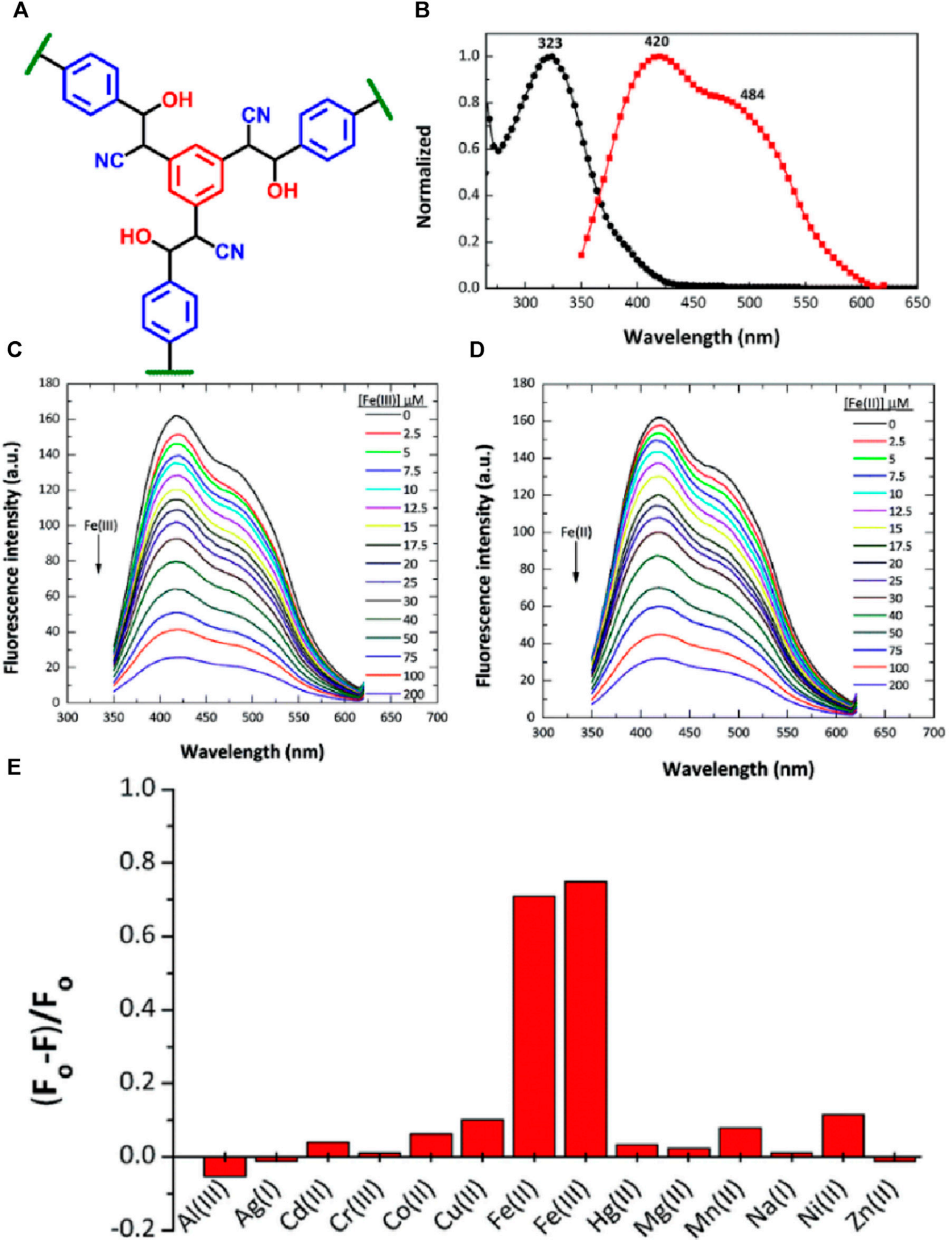
**(A)** The structure of COP-100; **(B)** The UV-visible absorption and fluorescence emission of COP-100 measured in DMF solution. The changes in fluorescence emission of COP-100 upon the incremental addition of a solution of **(C)** Fe(III) and **(D)** Fe(II) ions in DMF. **(E)** Selectivity plot for sensing ability of COP-100 towards Fe(II) and Fe(III) ions over other metal cations. Reprinted with permission from (Özdemir et al., 2015). Copyright 2015 Royal Society of Chemistry.

In the year 2015, a new class of covalent organic polymer (COP-100) was specifically designed for the selective detection of Fe(III) and Fe(II) ions (Özdemir et al., 2015) (Figure 2A for structure). COP-100 is functionalized with nitrile (-C≡N) functional groups for task-specific sensing applications (Sun et al., 2020). Interestingly, due to the incomplete conjugation, COP-100 displayed a higher absorption value at λ = 323 nm compared to other cyano-containing poly(*p*-phenylene vinylene compounds (Liu et al., 2009). COP-100 also showed a strong Stokes shift of 715 cm^–1^, resulting from intramolecular interactions among the aryl units. COP-100 was employed as an excellent sensor for Fe(II) and Fe(III) ions with capabilities to sense under both acidic and neutral conditions. However, the efficiency of quenching was slightly reduced to approximately 24% at pH levels of 10.5 and 14. The emission spectra of COP-100 showed a peak at λ = 420 nm (Figure 2B), which is attributed to the ortho-intramolecular exchanges among the aryl units, and the strong fluorescence emission of COP-100 was quenched significantly upon the addition of Fe(II) and Fe(III) ions in DMF solution (Figures 2C, D), while the addition of other competing metal ions such as Al(III), Ag(I), Cd(II), Co(II), Cr(III), Cu(II), Hg(II), Mg(II), Mn(II), Na(I), Ni(II), and Zn(II) ions showed almost no to poor fluorescence quenching (Figure 2E). The selective sensing of Fe(III) and Fe(II) ions by COP-100 was found to be highly sensitive even at low concentrations. A good linear plot was obtained from the fluorescence titration studies and the Stern–Volmer fluorescence quenching constant (*K*_SV_) was calculated from the slope of the linear curve as 2.58 × 10^4^ M^–1^ for Fe(II) and 2.97 × 10^4^ M^–1^ for Fe(III). Remarkably, COP-100 exhibited excellent fluorescent quenching responses towards Fe(II) and Fe(III) ions, with an impressive limit of detection (LoD) of 2.13 × 10^−7^ M and 2.45 × 10^−7^ M, respectively. These results demonstrate the significance of COP-100 as a highly effective fluorescent sensor for selective and sensitive detection of Fe(II) and Fe(III) ions.

**FIGURE 3:**
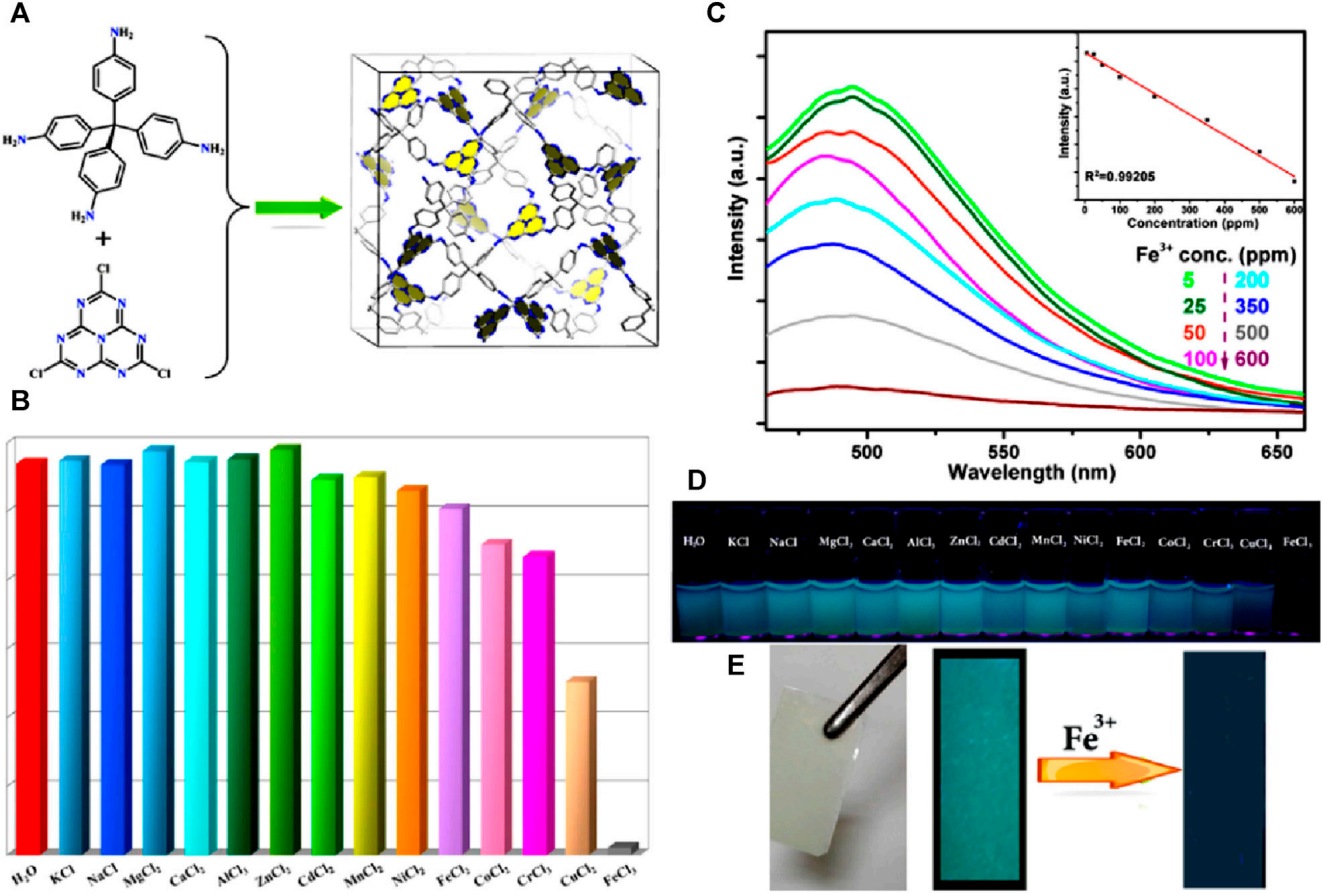
**(A)** One-step synthesis of POP-HT; **(B)** The relative changes in emission intensities of POP-HT dispersed in acidic aqueous solutions in the presence of different metal cations; **(C)** The gradual quenching of fluorescence intensity of POP-HT upon mixing Fe(III) ions in increasing concentrations (Inset: a plot of changes in intensity vs*.* concentration of Fe(III) ions); **(D)** Colorimetric photographs of POP-HT after the addition of different metal cations imaged under UV lamp; **(E)** Photograph of POP-HT thin film imaged under natural light and UV lamp before and after the addition of Fe(III) ions. Reprinted with permission from (Ma et al., 2016). Copyright 2016 American Chemical Society.

A new fluorescent POP, namely, POP-HT, capable of both quantitative and qualitative detection of Fe(III) ions was reported by Feng’s group (Ma et al., 2016). POP-HT offers two distinct advantages, firstly the presence of open Lewis base sites within the pores, and secondly, the extended π-system of s-heptazine, which contributes to POP-HT’s luminescent nature (Figure 3A). These unique characteristics make POP-HT an intriguing material for metal ions sensing studies. In the selectivity experiment, alkali, alkaline-earth, and transition metal ions with filled d-shells did not exhibit any noticeable changes in luminescence behavior. However, slight fluctuations were observed with certain transition metals, including Co(II), Ni(II), Fe(II), Fe(III), Cu(II), and Cr(III) ions (Figure 3B). Among these transition metals, Fe(III) ions demonstrated nearly complete luminescence quenching of POP-HT within a short exposure time (Figure 3C); this is due to the formation of strong coordination bonds between Fe(III) and available heterocyclic nitrogen atoms. The selective sensing of Fe(III) ions by POP-HT is also reflected by noticeable color changes both in solution and in thin film (Figures 3D, E). To gain a deeper understanding of the mechanism, further investigations were conducted to analyze the bonding interactions between Fe(III) ions and nitrogen atoms within the POP-HT framework. To ascertain the potential binding sites, calculations using the Gaussian-03 package at the B3LYP/def2-TZVPP level were performed to determine the bond distance and interaction energy between Fe(III) ions and different nitrogen atoms. The interaction energy calculations revealed intriguing findings regarding the bonding of Fe(III) ions to nitrogen atoms within the s-heptazine as well as NH groups of POP-HT. The interaction energy for the bonding of Fe(III) ions with nitrogen atoms of the s-heptazine groups was approximately −35.1 kcal/mol, indicating a significantly more favorable interaction compared to bonding with nitrogen atoms of the NH groups, which showed an interaction energy of −15.5 kcal/mol. These theoretical results provide a deeper understanding of the specific interactions between Fe(III) ions and the nitrogen atoms within POP-HT. This information is further supported by experimental XPS data. The XPS spectra analysis revealed significant shifts in the nitrogen atoms of POP-HT upon the introduction of Fe(III) ions. Among all the nitrogen atoms in the material, the most pronounced shift was observed in the N1s spectra. Specifically, the bonding energy of the N1s peak in Fe(III)@POP-HT shifted from 399.18 eV to 400.07 eV, exhibiting an upshift of 0.89 eV compared to free POP-HT. This substantial shift in energy provides compelling evidence for the presence of exceptionally strong interactions between Fe(III) ions and the nitrogen atoms within the POP-HT framework. The combined experimental and computational evidence offers valuable insights about the mechanism of sensing Fe(III) ions. In addition to its luminescence quenching ability, the POP-HT sensor also exhibited luminescence enhancement when exposed to the solvent 1,4-dioxane. This effect is attributed to hydrogen bonding between the H-atom of the imino group in the sensor and the O-atom of 1,4-dioxane. This multifunctional luminescence sensor, therefore, demonstrates practical applications for sensing Fe(III) ions and the hazardous compound 1,4-dioxane, even in an aqueous medium.

**FIGURE 4:**
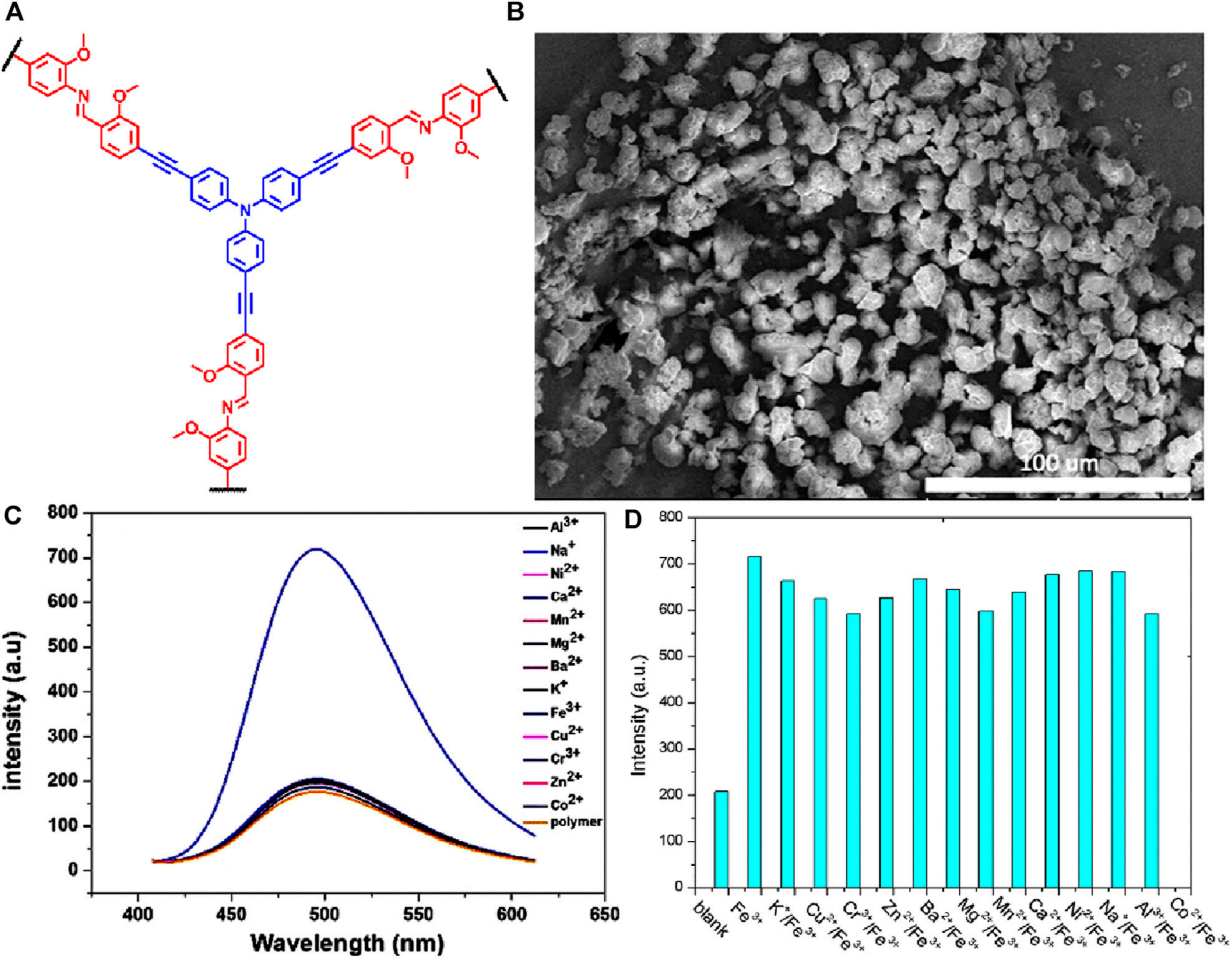
**(A)** The structure of polymer TPA-COP and **(B)** its SEM image; **(C)** The selective fluorescence sensing of TPA-COP for Fe(III) ions over other metal cations; **(D)** competitive sensing plot. Reprinted with permission from (Guo et al., 2018). Copyright 2018 Springer.

An interesting triphenylamine (TPA) scaffold-based covalent organic polymer (TPA-COP) was designed for the selective detection of Fe(III) ions in solution (Guo et al., 2018) (Figure 4A for structure). TPA-COP exhibited intriguing properties and can serve as a highly efficient fluorescent sensor for the selective and sensitive detection of Fe(III) ions, with a remarkable detection limit of 10^–7^ mol/L. Fluorescence sensors with Schiff base receptor sites are well-established due to their exceptional characteristics, including adjustable electronic and photophysical properties, strong chelating capabilities, and exceptional chemical stability (Afrin et al., 2023). The SEM imaging of as-synthesized TPA-COP showed an amorphous morphology composed of aggregates of larger particles (Figure 4B). The TPA-COP structure, with its extended π-conjugation and tridentate-coordinated Schiff base, facilitates the efficient sensing of Fe(III) ions. Experimental investigations demonstrated a turn-on fluorescence sensing property upon the introduction of Fe(III) ions to the TPA-COP solution, while the mixing of other metal ions showed almost no changes in fluorescence emission of TPA-COP in the THF solution (Figure 4C). The observed high selective sensing of Fe(III) by TPA-COP was also supported by the competitive fluorescence titration studies in the co-existence of other competing metal cations (Figure 4D). This remarkable sensing ability of TPA-COP was attributed to the strong coordination of the tridentate chelating Schiff base sites with Fe(III) ions, which results in the inhibition of PET from the imine nitrogen to the excited state of TPA-COP and thus, eventually causes the chelation-induced enhanced fluorescence effect. Confirmation of the coordination interaction between TPA-COP and Fe(III) ions was achieved through various analytical techniques, FT-IR, NMR spectroscopic analysis, and fluorescence lifetime experiments. For instance, the ^1^H NMR analysis of the 1:1 mixture of the TPA-COP and Fe(III) ions in DMSO-d_6_ displayed broadened and shifted signals, which are indicative of the presence of a paramagnetic iron center. Specifically, the signal corresponding to the imine proton initially observed at *δ* = 8.74 ppm, experienced a downfield shift to *δ* = 7.38 ppm upon the addition of Fe(III) ions. This shift towards higher chemical shift values can be attributed to the binding of Fe(III) ions to the imine site. The observed broadening and shifting of signals, particularly for the imine proton and the methoxy groups, provide evidence of the coordination of Fe(III) ions with the imine site and the associated changes in the chemical environment surrounding the methoxy groups. In the FTIR spectra of a 1:1 mixture of the TPA-COP with Fe(III) ions, the stretching vibrations associated with the imine group were observed at lower wave numbers compared to those of the TPA-COP alone. This shift towards lower wave numbers is consistent with the metal-ligand coordination interactions between heteroatom and Fe(III). To investigate the effect of Fe(III) on the luminescence intensity, time-resolved fluorescence studies were conducted. The fluorescence lifetime of TPA-COP in tetrahydrofuran (THF) was determined to be 1.26 ns. Upon the addition of Fe(III) ions solution, the fluorescence lifetime of TPA-COP slightly increased to 1.32 ns. This indicates that the presence of Fe(III) ions has a notable influence on the fluorescence lifetime of the sensor. These characterization techniques provide valuable evidence supporting the proposed sensing mechanism and the successful coordination of Fe(III) ions with the tridentate sites on TPA-COP.

**FIGURE 5:**
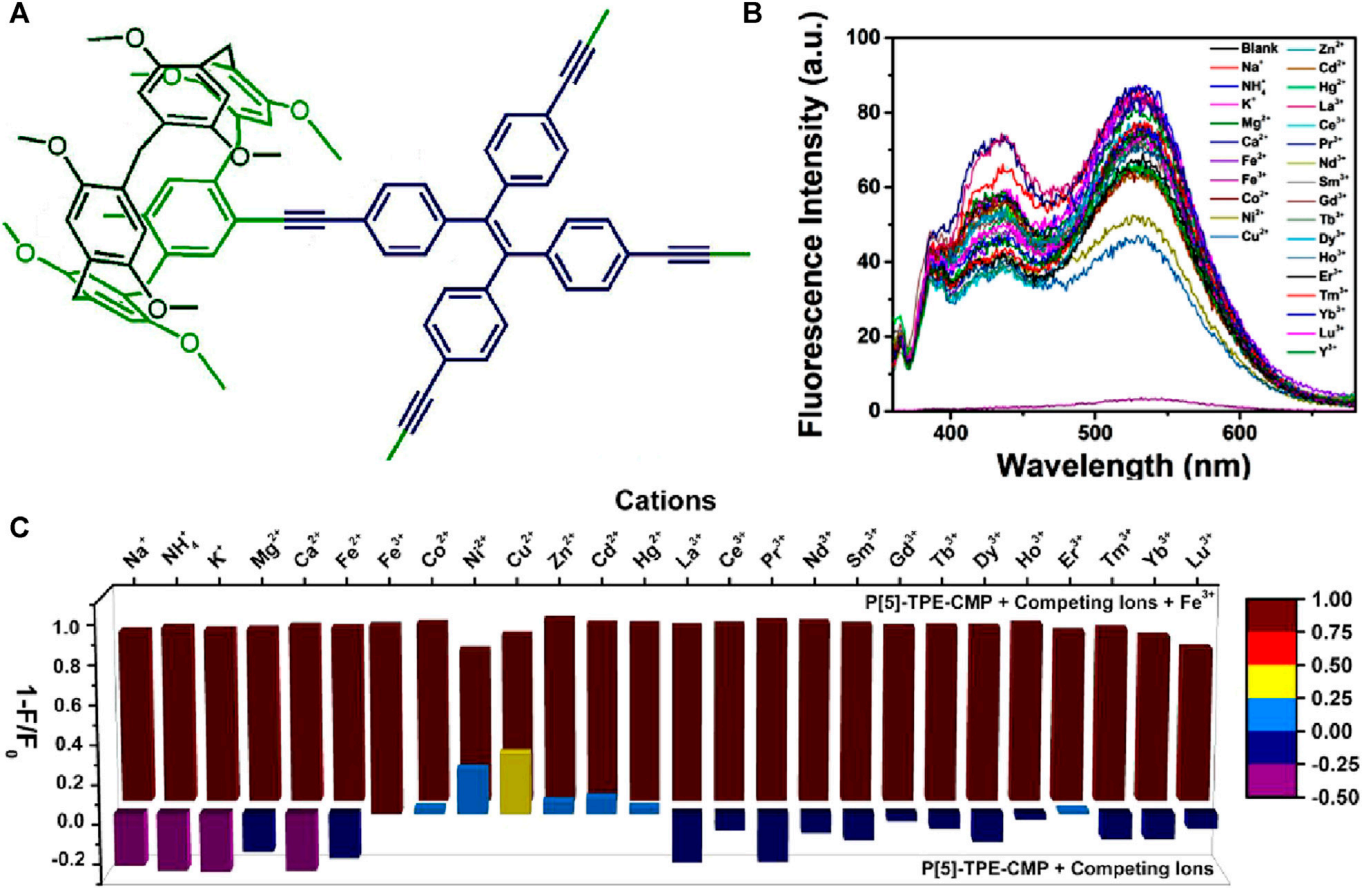
**(A)** The structure of polymer P[5]-TPE-CMP; **(B)** The selective fluorescence sensing of P[5]-TPE-CMP for Fe(III) ions over other metal cations; **(C)** Fluorescence quenching degrees (1-F/F_0_) in competition experiments of P[5]-TPE-CMP. Front: P[5]-TPE-CMP with different competing cations; back: P[5]-TPE-CMP with Fe(III) and the same equivalent of cations. Reprinted with permission from (Li et al., 2018). Copyright 2018 Willey.

In the year 2018, Yang et al. introduced a novel strategy for enhancing the fluorescence sensing performance of COPs by designing macrocycle-derived porous organic polymers (Li et al., 2018). The installation of macrocyclic structures within the polymeric networks improves the photophysical properties of both polymers and macrocycles by preventing the commonly observed molecular aggregation in solution (Skorjanc et al., 2021). In their work, Yang et al. have successfully developed a novel conjugated microporous polymer, P[5]-TPE-CMP, by linking pillar[5]arene macrocycle with tetraphenylethylene (Figure 5A) (Li et al., 2018). This P[5]-TPE-CMP polymer exhibited remarkable sensing capabilities for Fe(III) ions through an aggregation-induced emission (AIE) mechanism. The unique combination of a macrocyclic host system and solid porous polymer properties provides several advantages, including recyclability, insolubility, and so on. The presence of pillar[5]arene rings within the polymer plays a crucial role in both the luminescent properties and ion recognition abilities of the covalent organic framework. The pillar[5]arene cavity within the polymer contributes to the occurrence of two-photon fluorescence (TPF), as evidenced by a comparison of the emission spectra of the polymer with and without pillar[5]arene counterpart. Furthermore, the presence of a tetraphenylethylene (TPE) unit in the polymer influences the fluorescence properties by inducing an AIE effect, which is responsible for the fluorescence observed in the system. Fluorescence titration experiments revealed that the most significant quenching effect occurred upon the addition of Fe(III) ions, with a quenching efficiency of 92.9% (Figures 5B, C). This strong quenching can be attributed to the size-matching effect between the pillar[5]arene cavity and Fe(III) ions. X-ray photoelectron spectroscopy (XPS) data further supported the size-matching effect and highlighted the specific interaction between the methoxy group of pillar[5]arene and Fe(III) ions. Additionally, the reversibility of the sensing process was achieved by washing the P[5]-TPE-CMP polymer with water and subsequent centrifugation, as the polymer demonstrated high stability and insolubility. In addition to sensing Fe(III) ions, this polymer also can sense 4-amino azobenzene, an organic dye with carcinogenic properties, through a fluorescence quenching mechanism. These findings highlight the potential of conjugated macrocycle polymers as TPF sensors for the detection of ions and organic molecules. The rational design and fabrication of smart sensing devices based on these polymers have a significant impact on the field of sensing.

**FIGURE 6:**
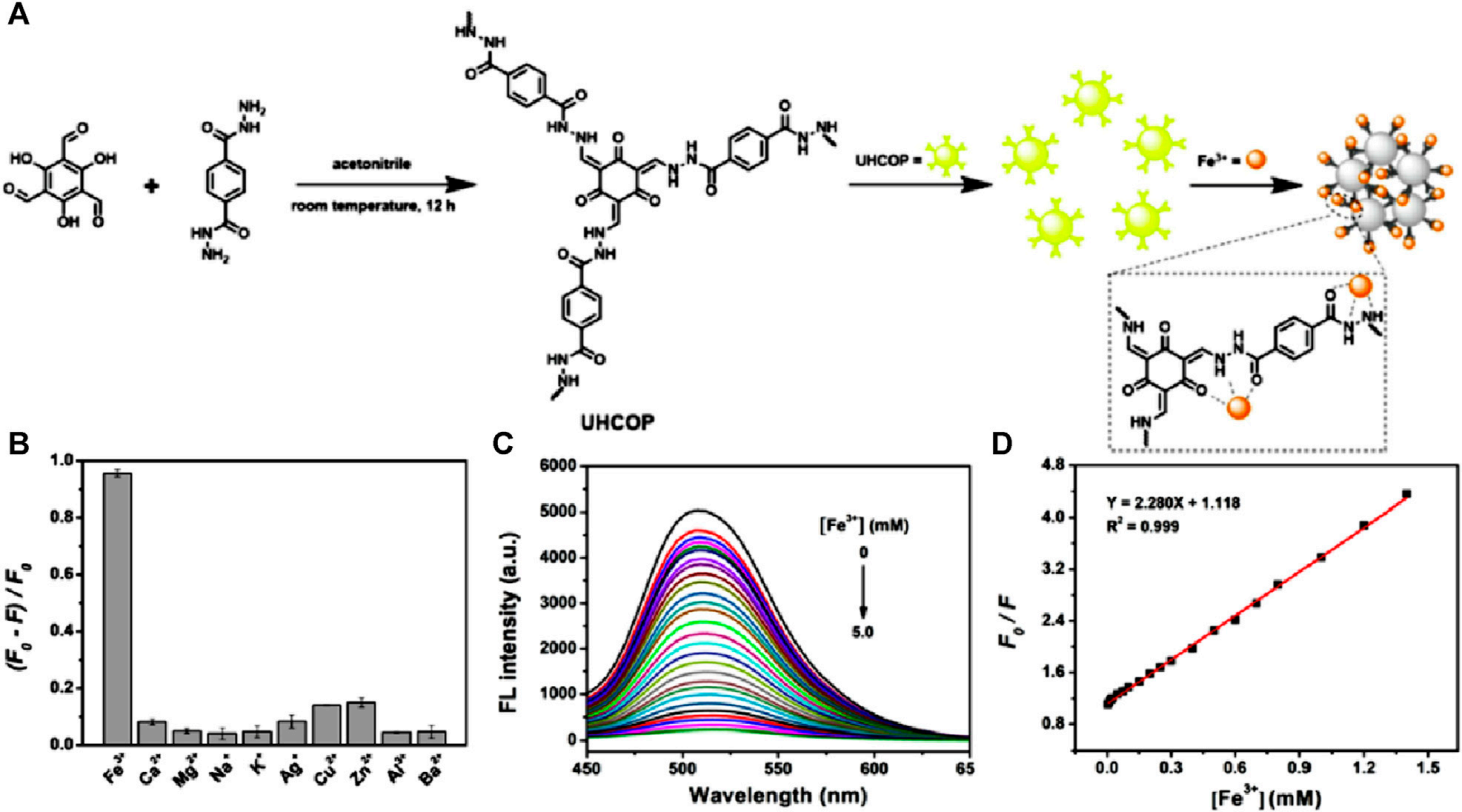
**(A)** Schematic representation of the synthesis of UHCOP and its subsequent sensing of Fe(III) ions in aqueous solution. **(B)** Selectivity plot showing the preferential binding of Fe(III) by UHCOP. **(C)** The observed fluorescence quenching for UHCOP after the addition of Fe(III) ions and **(D)** corresponding Stern–Volmer plot. Reprinted with permission from (Ji et al., 2022). Copyright 2022 Elsevier.

Application of COPs-based fluorescence sensors for the detection of Fe(III) ions in aqueous medium is still an unexplored territory because of their undesired fluorescence performance shown by the large COP particles as well as the low stability and weak emission of COPs in aqueous medium (Tashvigh and Benes, 2022). In 2022, Wang et al reported an ultra-small hydrazone-linked covalent organic polymer (UHCOP) was synthesized by using a simple Schiff base reaction between 2,4,6-trihydroxy-1,3,5-benzenetricarbaldehyde and 1,4-benzenedicarbohydrazide in acetonitrile solvent at room temperature (Figure 6A) (Ji et al., 2022). The average size of UHCOP analyzed using DLS results was found to be in the range of 7.98 ± 0.97, giving high dispersibility in the aqueous medium and more surface area for the polymer due to its extra small size. High hydrothermal stability as well as high dispersibility in an aqueous medium made the polymer UHCOP a promising probe for the detection of Fe(III) ions. UHCOP exhibited a strong and stable fluorescence emission at λ = 510 nm when excited at λ = 362 nm and was employed as a sensor for the detection of Fe(III) ions in aqueous solution. The synthesized polymer UHCOP upon fluorescent titrations with various cations gave the highest amount of quenching for Fe(III) ions (Figure 6B). The large quenching of emission intensity by Fe(III) ions is caused by coordination interaction which resulted in aggregation-caused quenching (ACQ) (Figures 6C, D). Sensor UHCOP was found to be highly stable, having fast response time (within 2 min), showed high selectivity, and excellent sensitivity (LoD = 2.5 µM), and was even found to be effective in real water samples. This study presented a simple method for the synthesis of ultra-small COPs for fluorescence-based sensing in aqueous solutions and showcased the capability of the synthesized polymer UHCOP as a reliable sensor system for the detection of Fe(III) ions in water samples.

**FIGURE 7:**
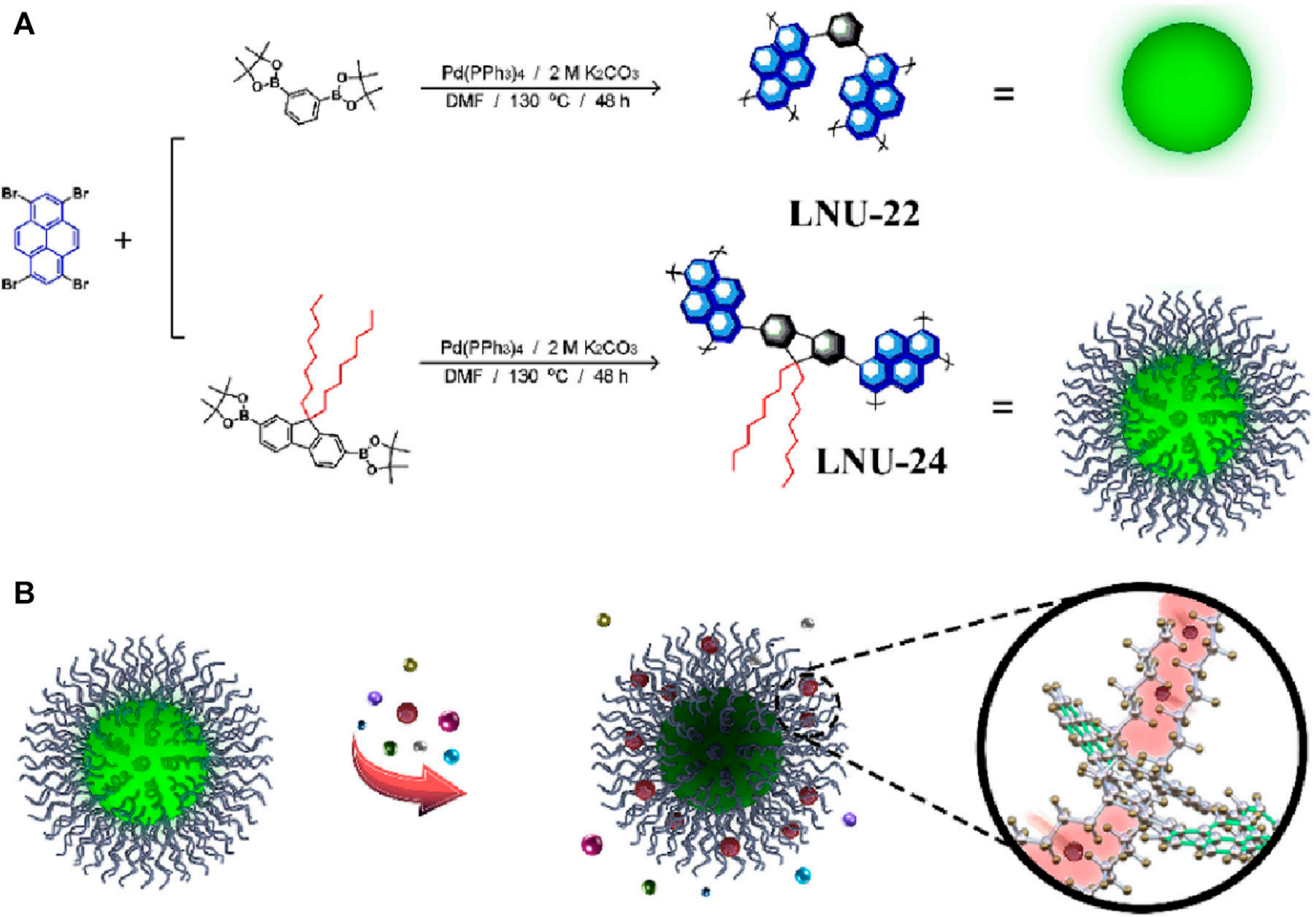
**(A)** Schematic representation of the synthesis of LNU-22 and LNU-24. **(B)** Fluorescence sensing of Fe(III) by LNU-22 and LNU-24. Reprinted with permission from (Yan et al., 2022). Copyright 2022 Wiley.

While being widely used as a fluorescent sensor, pyrene and its derivatives suffer from a decrease in fluorescence quantum yield due to the tight packing of porous solids affects the practical applicability of such sensor (Feng et al., 2023). In their work, the Xia group innovatively introduced long-chain alkanes into a porous aromatic network which resulted in the improvement of the quantum yield to a great extent (0.79% (pure phenyl-based PAF) to 22.98% (long-chain alkane-grafted PAF) (Yan et al., 2022). This porous aromatic framework was used successfully as a fluorescent sensor for Fe(III) with high selectivity, sensitivity, and high quantum yield efficiency. Two sets of porous frameworks LNU-22 & LNU-24 were prepared by coupling 1,3,6,8-Tetrabromopyrene with two different moieties through Suzuki coupling reactions (Figure 7). LNU-22 was a pure phenyl-based system prepared using 1,3-phenylboronic acid bis(pinacol) ester, while LNU-24 included a long alkyl chain to the architecture using 9,9-dioctylfluorene-2,7-diboronic acid bis(pinacol) ester (Figure 7). While comparing the FTIR spectra of LNU-22 & LNU-24 with that of monomers, the absence of B-O (at 1,368 cm^−1^) and C-Br (at 495 cm^−1^) stretching bands suggested the successful completion of Suzuki coupling reactions. In addition to this, LNU-24 contained an intense peak corresponding to the C−H vibration (at 2,930 cm^−1^). ^13^C NMR revealed distinctive peaks in the *δ* = 115–155 ppm range were associated with the aromatic carbon atoms of both the architectures and additional chemical shifts of *δ* = 20–40 ppm ascribing to the alkyl chains were found in LNU-24. LNU-24 possessed greater thermal stability, being stable up to 350°C, and was insoluble in most common solvents, thus exhibiting high chemical stability as well. PXRD and SEM analysis of the PAFS revealed them to be made up of erratically stacked nanospheres, the majority of which have a diameter of 0.2–1 μm. It was found that LNU-22 and LNU-24 have surface areas of 524 and 71 m^2^ g^−1^, respectively. Due to the occupation of long-chain alkanes in the pore spaces inside the PAF architecture, LNU-24’s BET surface area was noticeably reduced. Under excitation at λ = 365 nm, LNU-22 was a blue-green color solid with maximum emission at λ = 480 nm, while LNU-24 was a yellow-green colored solid with emission maxima at λ = 508 nm. The fluorescent sensing response of both the LNUs was recorded in THF/H_2_O suspension and both gave an observable color change under UV light and selective quenching response for Fe(III) ions. LNU-24 yielded better quantum yield and better quenching efficiency than LNU-22. Careful investigation of the quenching mechanism revealed that, compared to other metal ions, only Fe(III) has a UV-vis absorption spectrum in the range of λ = 250–500 nm which overlaps with the UV-vis spectra of the LNUs. This causes the PAF sample and Fe(III) ions to compete for the light source energy through absorption, hence dimming the fluorescence. LNU-22 and LNU-24 gave a linear Stern–Volmer relation with *K*_SV_ values of 8.80 × 10^−2^ and 2.13 × 10^−3^ M^−1^ and high LoD values of 8.80 × 10^−2^ and 2.13 × 10^−3^ M. LNU-24 possess superior sensitivity compared to LNU-22 as the long alkyl chains capture the Fe(III) ions via ion/induced dipole interactions which enhances their competition efficiency.

## 4 Covalent organic frameworks (COFs)-based fluorescent sensors for sensing Fe(III) ions

**FIGURE 8:**
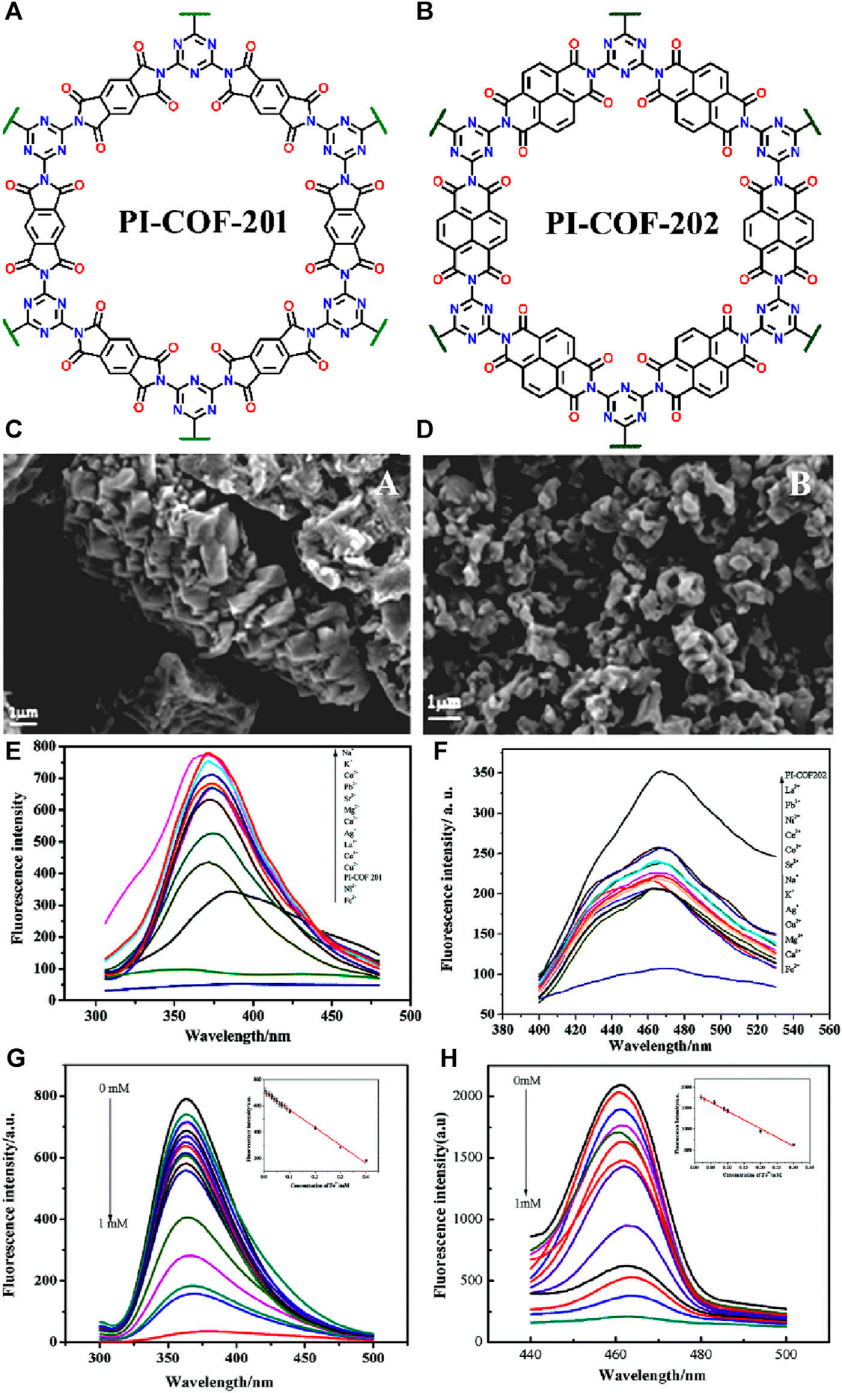
The extended polymeric structure of **(A)** PI-COF-201 and **(B)** PI-COF-202; **(C,D)** their corresponding SEM images showing different morphological features; The effect of different metal cations on the fluorescence emission of **(E)** PI-COF-201 and **(F)** PI-COF-202; The relative changes in emission intensity of **(G)** PI-COF-201 and **(H)** PI-COF-202 upon the incremental addition of Fe(III) solution. Reprinted with permission from (Wang et al., 2017). Copyright 2017 Royal Society of Chemistry.

In 2017, Wang et al. developed an eco-friendly synthetic protocol for developing two luminescent COFs, namely, PI-COF-201 and PI-COF-202, by simply heating melamine (MA) with pyromellitic dianhydride (PMDA) and naphthalene tetracarboxylic dianhydride (NTDA), respectively (Figures 8A, B for the structure of COFs) (Wang et al., 2017). Both PI-COF-201 and PI-COF-202 were employed as potential fluorescence sensors for selective detection of Fe(III) ions. The crystalline nature and well-defined structures of the two PI-COF materials were evident from their SEM images (Figures 8C, D). PI-COF-201 displayed a cone-shaped morphology indicating a specific arrangement of the constituent molecules within the framework (Figure 8C). On the other hand, PI-COF-202 exhibited a block-shaped morphology implying a different arrangement and packing of the building units compared to PI-COF-201 (Figure 8D). In addition, other characterization techniques such as XRD analysis, and thermogravimetric analysis supported the excellent crystallinity and the considerable thermal stability of COFs structures. Owing to their high dispersibility, all the fluorescence sensing studies were performed in DMF solution. When the suspension of PI-COF-201 and PI-COF-202 were exposed to various metal ions such as Na(I), K(I), Co(II), Pb(II), Sr(II), Mg(II), Ca(II), Ag(I), La(III), Ce(III), Cu(II), Fe(III), and Ni(II) ions, an interesting fluorescence sensing responses were observed. All other metal ions showed fluorescence enhancement with a blue shift in emission maxima, but Fe(III) and Ni(II) ions exhibited quenching of fluorescence emission intensity of COFs (Figures 8E, F).

Notably, Fe(III) ions elicited the largest fluorescence quenching with both the COF sensors. From the fluorescence titration profile, the *K*_SV_ was determined to be 3.23 × 10^−3^ M^–1^ for PI-COF-201 (Figure 8G) and 3.54 × 10^3^ M^−1^ for PI-COF-202 (Figure 8H). All other metal ions, except Fe(III) ions, did not show any new absorption peak which indicates that there is certainly no energy transfer process occurring from the emission level of COFs to the metal energy levels, while in the case of Fe(III) ions, an intense new absorption band was observed which revealed the existence of powerful energy transfer between the excitation state of COFs to the unfilled d orbitals of Fe(III) ions. This energy transfer mechanism facilitated the development of turn-off Fe(III) sensors with exceptional selectivity and sensitivity. Furthermore, the N atoms present on the COF pore walls form an effective coordination bonding with the electron-deficient Fe(III) ions, contributing to the excellent quenching behavior observed. This coordination interaction further enhanced the sensing capability of COFs towards Fe(III) ions. In summary, the innovative synthetic approach employed in the fabrication of PI-COF-201 and PI-COF-202, coupled with the specific interactions between Fe(III) ions and the COF structures, resulted in a highly selective and sensitive turn-off fluorescence sensors for Fe(III) ion detection.

**FIGURE 9:**
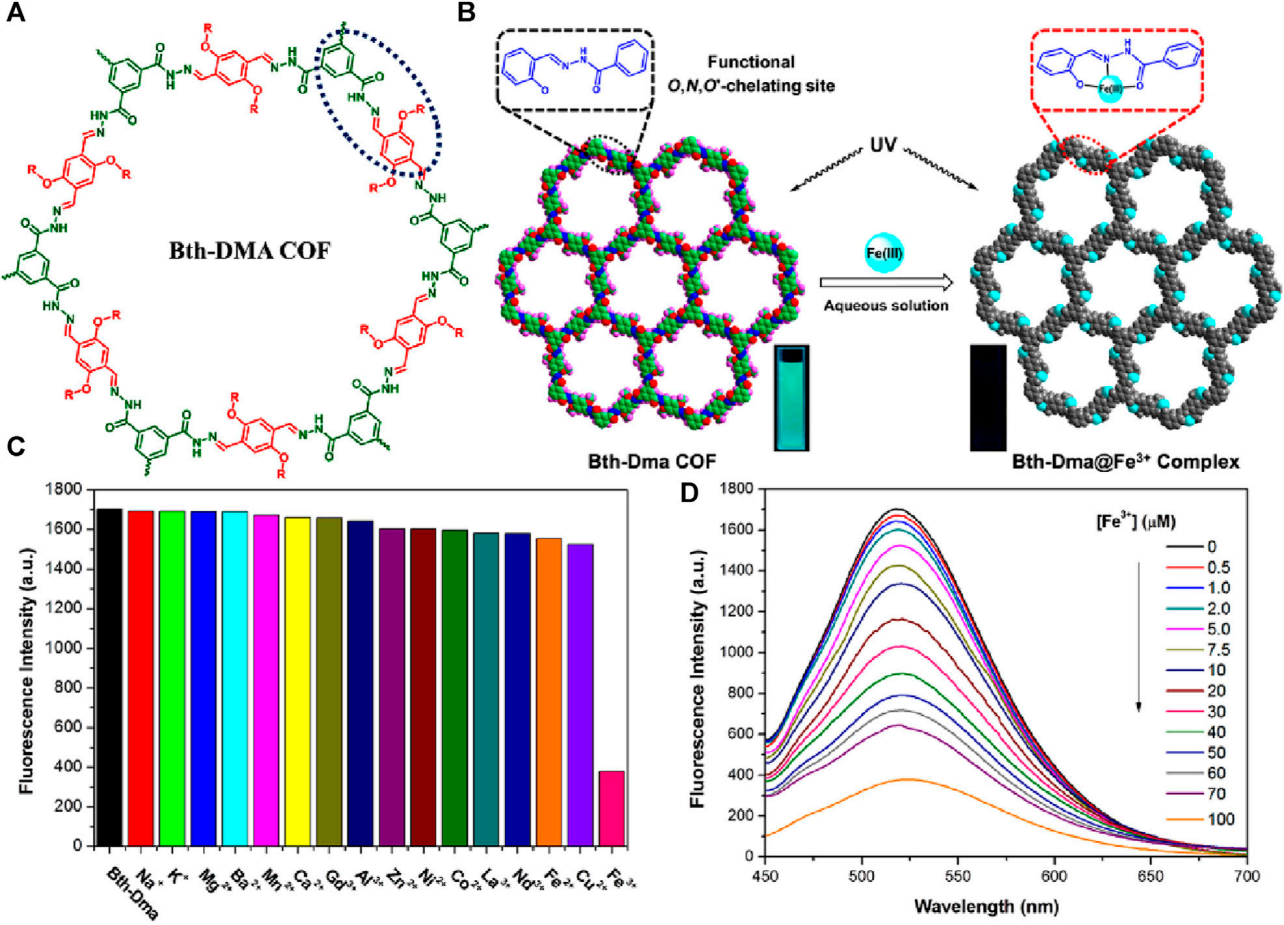
**(A)** The structure of hydrazone-linked covalent organic framework Bth-Dma; **(B)** The proposed mode coordination of Bth-Dma sensor with Fe(III) ions (Inset: observed turn-off colorimetric photographs). **(C)** The selective fluorescence sensing of Bth-Dma for Fe(III) ions over other metal cations; **(D)** The observed fluorescence quenching upon the incremental addition of Fe(III) ions solution. Reprinted with permission from (Chen et al., 2019). Copyright 2019 American Chemical Society.

In their work, Zhang et al. successfully synthesized a luminescent COF, namely, Bth-Dma, using condensation reactions of benzene-1,3,5-tricarbohydrazide (*B*th) with 2,5-dihydroxyterephthalaldehyde (Dha) (Chen et al., 2019) (Figure 9A). This COF (Bth-Dma) incorporates a predesigned O, N, O′-chelating unit, which imparts remarkable selectivity towards Fe(III) ions. The selective sensing of Fe(III) ions arises from its strong coordinating ability with O, N, and O′-chelating sites located within the pore wall of Bth-Dma (Figure 9B). The selective and strong binding of Fe(III) ions with Bth-Dma is also indicated by a sharp visual color change (Figure 9B inset). This coordination interaction is believed to influence fluorescence quenching by affecting energy or electron transfer involving excited states with d-orbital electrons. This coordination interaction was confirmed through thorough analysis using ^1^H NMR and X-ray photoelectron spectroscopy (XPS).

Firstly, the Bth-Dma@ Fe(III) complex was synthesized by immersing Bth-Dma COF powder in a FeCl_3_ aqueous solution for 2 days at room temperature. The resulting complex was collected through centrifugation, washed with water and THF, and then subjected to PXRD and XPS measurements. The PXRD pattern of the obtained Bth-Dma@ Fe(III) complex closely resembles that of the parent Bth-Dma COF, indicating the retention of the 2D COF structure. Additionally, the Fe 2p XPS spectrum of the Bth-Dma@ Fe(III) complex exhibits peaks at 711.4 and 724.8 eV, corresponding to the Fe 2p_3/2_ and Fe 2p_1/2_ binding energies, respectively. This further confirms the successful immobilization of Fe(III) ions within the pore channels of Bth-Dma COF. The N 1s peak in the hydrazone units of Bth-Dma COF also experiences a shift from 400.143 to 400.6 eV upon the addition of Fe(III) ions, supporting the occurrence of a binding event between the O, N, O′-chelating sites and Fe(III) ions in the resulting Bth-Dma@ Fe(III) complex. In the low concentration range of Fe(III) ions, a linear Stern–Volmer quenching was observed with a *K*_SV_ value of 2.3 × 10^4^ M^−1^ (Figure 9C). The calculated detection limit for Fe(III) ions was 0.17 μM demonstrating the high sensitivity of Bth-Dma COF towards Fe(III) ions. Furthermore, the effects of pH and counter anions on the fluorescence of Bth-Dma were investigated. The results showed that neither the pH (in the range of 4–10) nor the presence of anions (such as NO_3_^−^, Cl^−^, Br^−^, and OAc^−^) had a significant impact on the fluorescence quenching efficiency (Figure 9D). This suggests that the selective sensing of Fe(III) ions by Bth-Dma COF is minimally affected by changes in pH or the presence of different anions. In addition to Bth-Dma, the researchers also developed another COF Bth-Dha by utilizing benzene-1,3,5-tricarbohydrazide (*B*th) and 2,5-dihydroxyterephthalaldehyde (Dha). However, unlike Bth-Dma, Bth-Dha does not exhibit any emission properties. This lack of luminescence in Bth-Dha is attributed to non-radiative decay through excited-state proton transfer. On the other hand, the restricted intramolecular bond rotation makes Bth-Dma is highly emissive. Despite the difference in luminescence behavior, both COFs display exceptional crystalline nature and exhibit robust chemical stability when exposed to various solvents.

**FIGURE 10:**
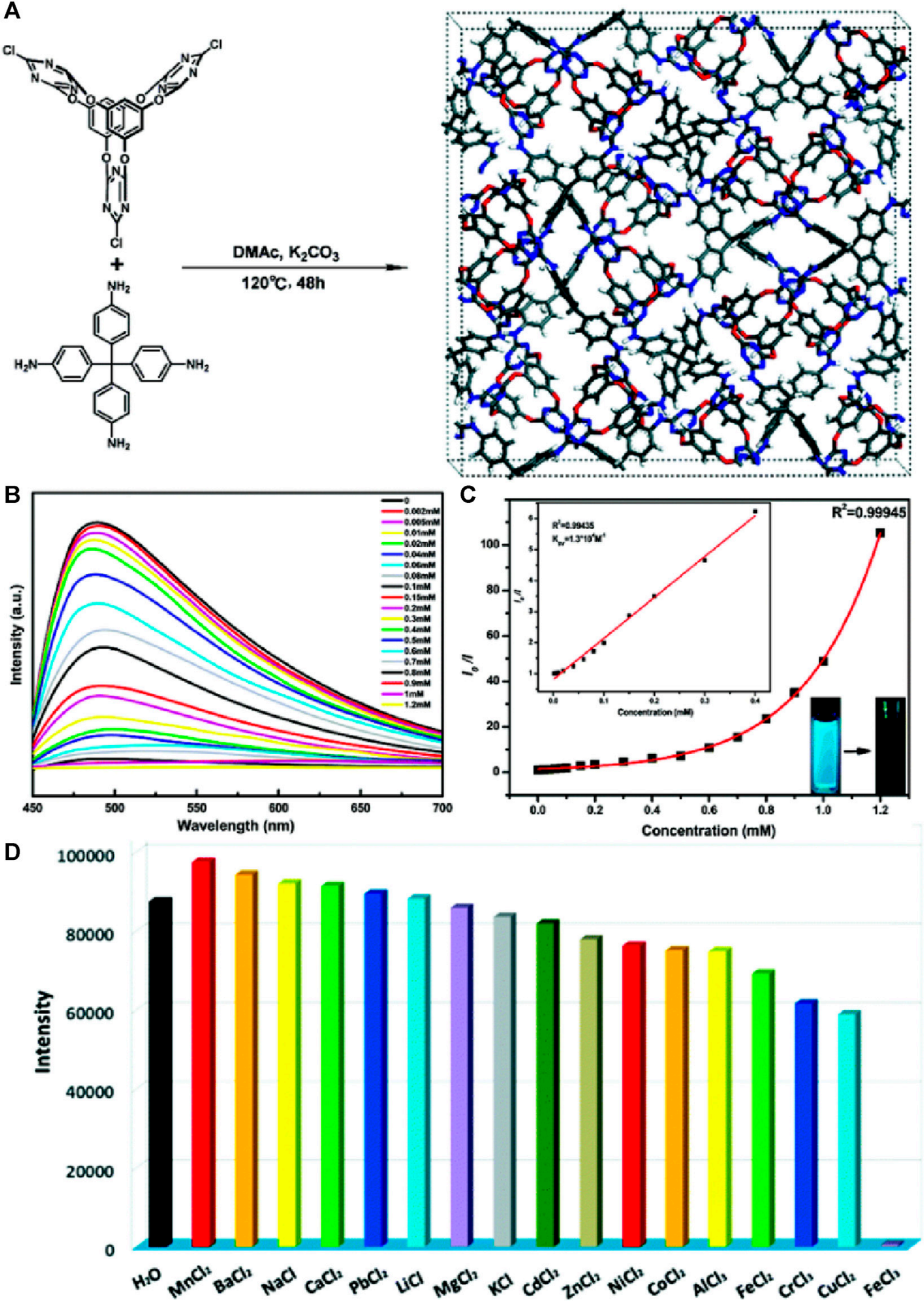
**(A)** Schematic representation of the synthesis of COF-TT; **(B)** The fluorescence emission spectra of COF-TT suspensions upon the addition of Fe(III) ions in an acidic aqueous solution and **(C)** its corresponding Stern–Volmer plot (inset: linear Stern–Volmer plot at low concentrations and visual color changes); **(D)** Relative fluorescence intensities of COF-TT dispersed in acidic aqueous solutions containing different competing cations. Reprinted with permission from (Li et al., 2019). Copyright 2019 Royal Society of Chemistry.

A novel three-dimensional luminescent covalent organic framework, COF-TT, was successfully synthesized via a solvothermal method by reacting a flexible core bis(tetraoxacalix[2]arene[2]triazine) and a rigid tetra(*p*-aminophenyl)methane (Li et al., 2019). COF-TT exhibited a three-dimensional structure with high porosity, allowing for efficient target analyte encapsulation (Figure 10A). The framework of COF-TT demonstrated exceptional fluorescence quenching capabilities against Fe(III) cations in aqueous media. Upon the addition of Fe(III) ions, a significant turn-off effect was observed, resulting in a drastic reduction of approximately 98.4% in the fluorescence intensity of COF-TT (Figure 10B). The primary reason for the decrease in fluorescence intensity was attributed to the host-guest interaction between the COF-TT framework and the ferric cations. The high sensitivity of COF-TT towards Fe(III) ions was demonstrated by calculating the detection limit, which was determined to be 3.69 × 10^−4^ M (Figure 10C).

The mechanism of quenching was elucidated based on three factors: i) the rupture of the framework skeleton, ii) the competition between cations and COF-TT for absorption, and iii) the robust host-guest chemistry between cations and COFs. To investigate the underlying mechanism, XPS and theoretical calculations were performed on COF-TT and various metal ions containing M^n+^@COF-TT. The XPS analysis showed that the O 1s peak of COF-TT shifted from 513.65 to 534.42 eV upon the addition of Fe(III) ions, indicating strong interactions between Fe(III) and O atoms. Similarly, the N 1s peak shifted to 399.94 eV from 399.18 eV, indicating enhanced bonding between Fe(III) and N atoms. In contrast, the interactions of other metal ions with O or N atoms were negligible, as evidenced by the lack of significant shifts in the corresponding XPS peaks. Theoretical calculations, employing the CAM-B3LYP method with the def2SVP basis set, supported the experimental findings. The optimized ground-state structures revealed Fe···O distances of 2.118 Å in the bis(tetraoxacalix[2]arene[2]triazine) core, Fe···N distances of 2.143 Å in the triazine unit, and Fe···N distances of 2.309 Å in the amine groups. The XPS analysis, theoretical calculations, and energy data indicate that the interaction of Fe(III) with O atoms (Fe···O) plays a significant role in fluorescence quenching in Fe(III)@COF-TT. Furthermore, COF-TT not only exhibits remarkable sensitivity to Fe(III) cations but also excellent quenching capabilities towards various anions, including CrO_4_^2−^, Cr_2_O_7_^2−^, and MnO_4_^−^, in aqueous media (Figure 10D). This unique feature allows for the detection and sensing of both cations and anions using a single COF system.

In 2021, Wang *et al* investigated the fluorescence sensing capabilities of a luminescent COF called TT-COF, which was synthesized through a Schiff base reaction between 2,5-dihydroxyterephthalaldehyde and 1,3,5-tris(4-aminophenyl)benzene (Zhang et al., 2021). This hollow spherical COF was chemically stable and possessed a high surface area of 1,500 m^2^ g^−1^. TT-COF upon dispersing in ethanol and then exciting at λ = 340 nm displayed a high emission peak at λ = 425 nm. The emission properties of the TT-COF mainly came from the π−π* transition in the conjugated system while the restricted rotation of the intra-molecular C=N bond further intensified the fluorescence (Li et al., 2020).

**FIGURE 11:**
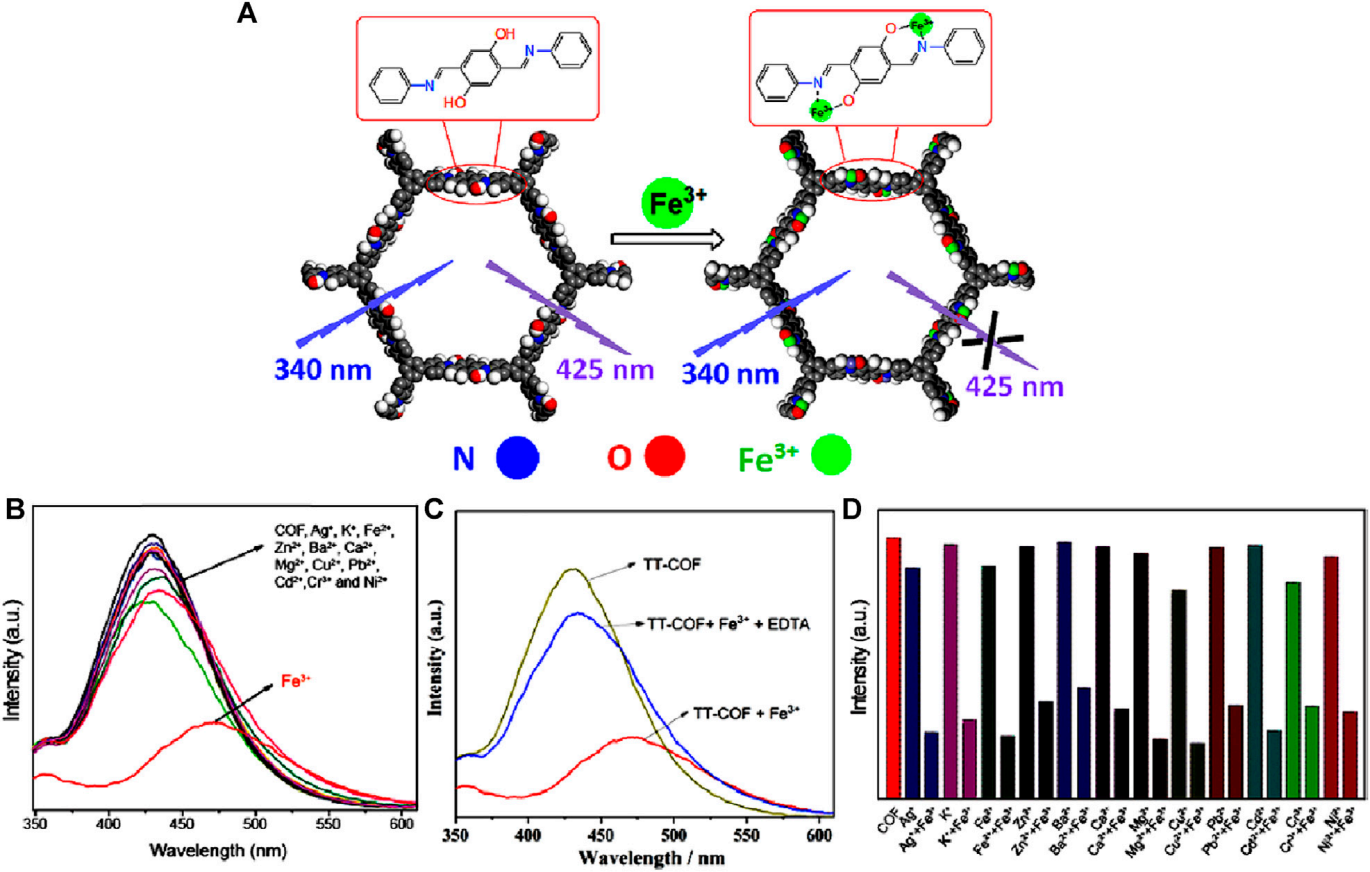
**(A)** Schematic representation of the mechanism of fluorescence-quenching-based sensing of Fe(III) by TT-COF. **(B)** The fluorescence emission spectra of TT-COF after the addition of different metal ions. **(C)** Reversible fluorescence sensing responses of TT-COF@Fe(III) before and after the addition of EDTA. **(D)** Bar diagram showing the high selectivity of TT-COF for Fe(III) detection. Reprinted with permission from (Zhang et al., 2021). Copyright 2021 Elsevier.

In fluorescence titration experiments conducted using TT-COF suspension in ethanol with various metal ions, it was observed that Fe(III) ions caused the most significant fluorescence quenching (Figures 11A, B, D). This effect was accompanied by a redshift of approximately 42 nm, attributed to the coordination bond formed between Fe(III) ion and N and O atoms, as well as ICT from the imine N atom to Fe(III). The distinctive framework and pore structure, i.e., the spatial arrangement of TT-COF acts as a barrier and prevents other metal ions from binding with it effectively giving the high selectivity for Fe(III) ions. Upon the addition of ethylenediamine tetraacetic acid disodium salt (EDTA) which has a strong chelating capacity, the fluorescence intensity was restored to the original state which also indicates the complex formation (Figure 11C). The mechanism of interaction was investigated using FT-IR and XPS. The FTIR spectrum of TT-COF@Fe(III) showed that the vibrational peaks for OH at 3,424 cm⁻^1^ was disappeared, and the C=N band at 1,613 cm⁻^1^ shifted to 1,622 cm⁻^1^. This confirms the participation of the O atom of OH and the N atom of C=N in the coordination. The XPS spectrum peaks observed at 723.93 eV and 711.83 eV in the spectrum of the TT-COF@Fe(III) complex represent the Fe 2p_1/2_ and Fe 2p_3/2_ binding energies, respectively. These findings suggest the effective binding of Fe(III) ions to the pore wall of TT-COF. The N 1s peaks attributed to the C-N and C=N binding energy were found to be present in both TT-COF and TT-COF@Fe(III). However, the peaks shifted from 401.44 eV to 398.88 eV–401.92 eV and 399.14 eV respectively indicating the coordination of Fe(III) ions with N atoms of the TT-COF framework (Li et al., 2019). Similarly, after the coordination of Fe(III) ions with TT-COF, there was a noticeable change in the O 1s peaks. The O 1s peaks of C-O and C-OH in TT-COF, originally at 534.86 eV and 532.55 eV, respectively shifted to 533.14 eV and 531.19 eV, following the coordination process (Li et al., 2019). Thus, the XPS spectrum also confirmed the involvement of N and O atoms of COF in the coordination process with Fe(III) ions by observing the alterations in binding energy in N 1s and O 1s spectra. A further understanding of the coordination mechanism was achieved through DFT calculations and UV-Vis absorption studies. UV-vis spectrums reveal that the excitation light needed for TT-COF fluorescence is competitively absorbed by Fe(III) ions, which lowers the excitation energy of TT-COF. The mechanism behind the fluorescence quenching effect of Fe(III) ions on TT-COF is due to absorption competition quenching and intra-molecular charge transfer.

**FIGURE 12:**
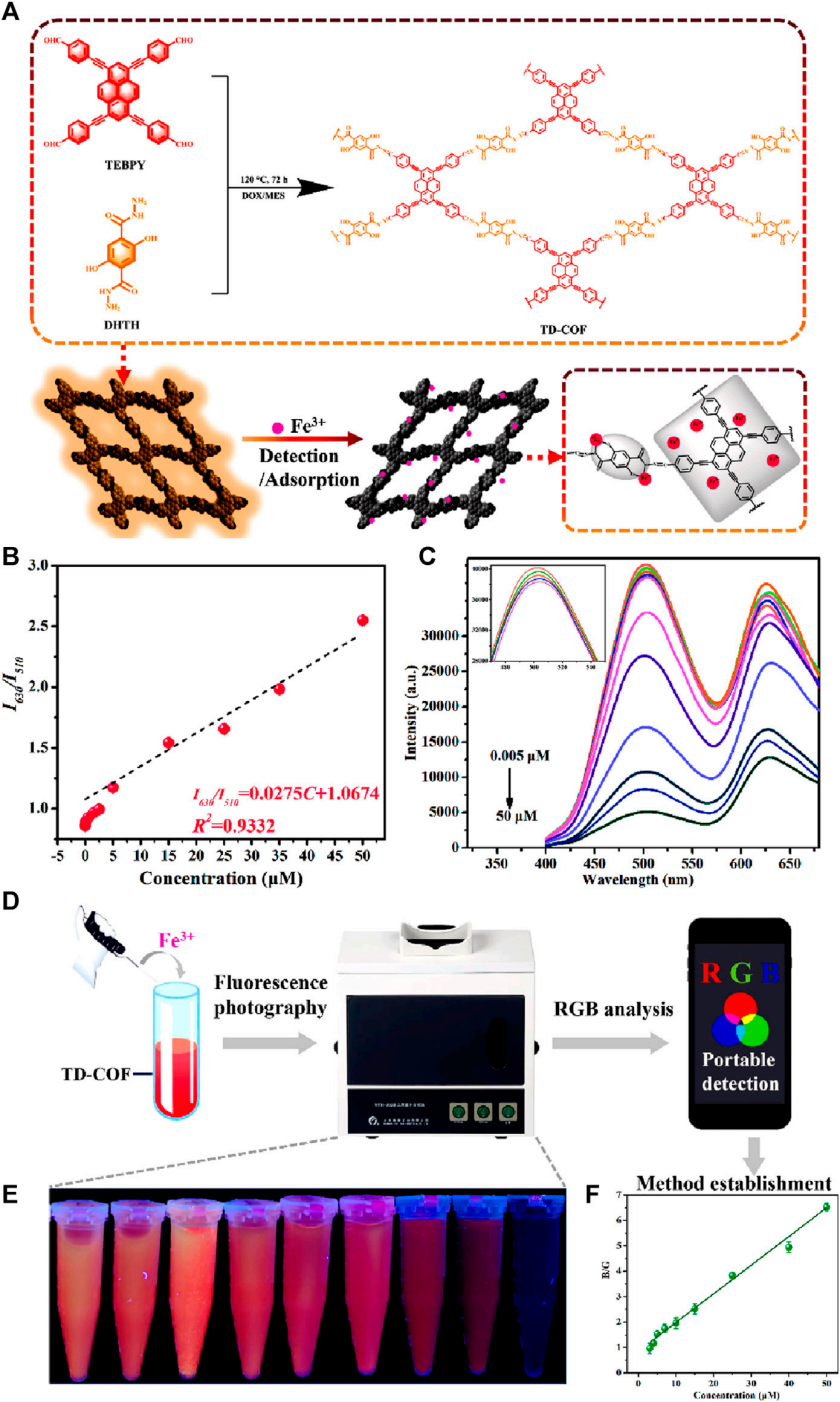
**(A)** Schematic representation of the synthesis of TD-COF and its application for the detection of Fe(III). **(B)** A plot of fluorescence intensity ratio (*I*_630_/*I*_510_) vs*.* concentration of Fe(III). **(C)** The changes in the fluorescence emission spectrum of TD-COF upon gradual addition of Fe(III). **(D)** Diagram of smartphone-integrated portable device and system for real-time monitoring Fe(III). **(E)** Fluorescence images of TD-COF in the presence of Fe(III) incubated with different concentrations. **(F)** The B/G value of TD-COF vs*.* Fe(III) concentrations. Reprinted with permission from (Zhang et al., 2023). Copyright 2023 Elsevier.

Recently, a highly efficient dandelion-like fluorescent COF was developed for ratiometric sensing and visual tracking of Fe(III) ions in aqueous suspension (Zhang et al., 2023). 1,3,6,8-tetrakis(4-ethynylbenzaldehyde)-pyrene (TEBPY) and 2,5-dihydroxyterephthalohydrazide (DHTH) functionalized with OH groups were taken as building blocks for the synthesis of dandelion-like TD-COF with a quantum yield of 36.4% and a dual emission at λ = 510 nm and 630 nm (Figure 12A).

The reason for the high quantum yield for TD-COF was assigned to hydrazone linkage and the high rigidity of TEBPY. The hydrazone linkage is known to overcome the non-radioactive decay of imine linkage. Functionalizing the OH site on the pore wall surface of TD-COF introduced rotational restriction through an intramolecular hydrogen bond. This led to the ESIPT effect, resulting in a green emission at λ = 510 nm. Additionally, the extended π-conjugation and enhanced planarity in TD-COF induced a red fluorescence emission at λ = 630 nm. Upon exploration of the ratiometric sensing of Fe(III) using TD-COF, a linear relation between *I*_630_/*I*_510_ and Fe(III) ions concentration with a linear fitting equation, I_630_/I_510_ = 0.0275C + 1.0674, along with the strong linear relationship (*R* ^2^ = 0.9332) was discovered with a theoretical limit of detection of 10.9 nM, which suggested the likelihood of static or dynamic quenching in the Fe(III)/TD-COF system (Figures 12B, C) (Chen et al., 2022). Computational calculations verified that the main process involved in the fluorescence quenching is PET transition from TD-COF to Fe(III) ions through metal-ligand coordination. The TD-COF also has an exceptional ability to adsorb the Fe(III) ions with an adsorption capacity at equilibrium (*q*m) of 833.3 mg/g owing to their robust interaction between TD-COF and Fe(III) which involves coordination between Fe(III) and unsaturated N, O atoms, as well as the cationic π-effect induced by conjugated π electrons. The selectivity studies showed that the fluorescence response (*I*_630_/*I*_510_) of TD-COF to Fe(III) (500 μM) was significantly higher compared to other ions at similar high concentrations. The presence of other interfering ions even at much higher concentrations also had either minimal or no impact at all on the specific recognition of Fe(III) by TD-COF. Using this framework TD-COF a smartphone-integrated ratiometric sensing apparatus was created to visually detect and track Fe(III) ions with a detection limit (Figures 12D–F) calculated as 2.81 µM which is less than the World Health Organization’s 5.36 μM limit for Fe(III) or the most recent EU drinking water quality directive 98/83/EC (3.6 μM/200 μg/L). Fe(III) was detected in actual samples, demonstrating the viability of the TD-COF technique with recoveries ranging from 97.41% to 102.27%. With its exceptional ratiometric detection and adsorption properties, TD-COF is a viable model for fluorescence sensing and visual Fe(III) ion monitoring.

**FIGURE 13:**
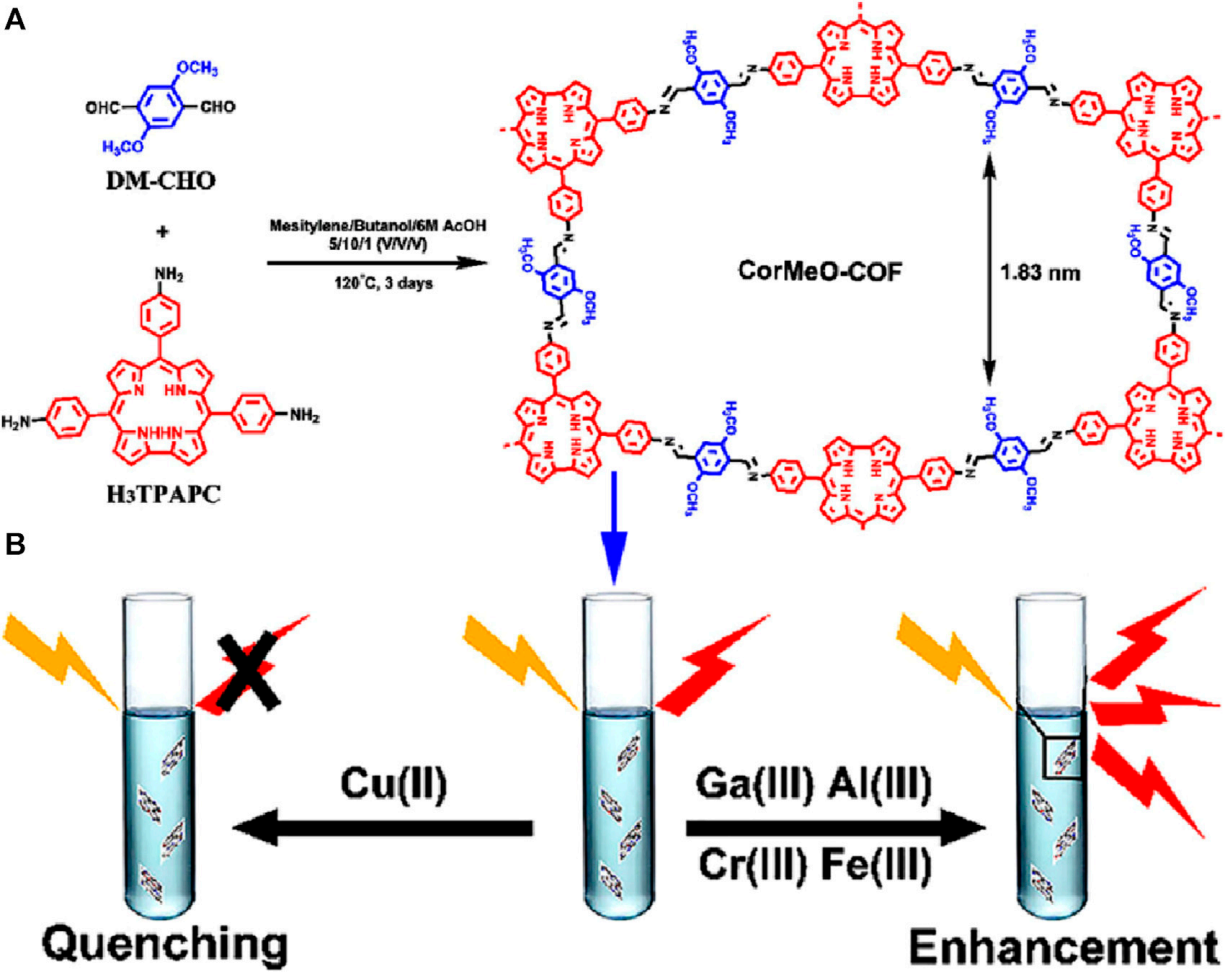
**(A)** Schematic representation of the synthesis of CorMeO-COF and **(B)** representation of its discriminative fluorescence sensing responses towards different metal ions. Reprinted with permission from (Li et al., 2020). Copyright 2020 American Chemical Society.

A novel corrole-based covalent organic framework, CorMeO-COF, was designed and successfully used for discriminative fluorescent sensing of different metal cations (Li et al., 2020). The 2-dimensional CorMeO-COF was synthesized via a [3 + 2] imine condensation reaction between C_2V_-symmetric 5,10,15-tris(p-aminophenyl)corrole (H_3_TPAPC) and 2,5-dimethoxy-terephthalaldehyde (DM-CHO) (Figure 13A). The unusual configuration of the corrole monomer led to the CorMeO-COF having an unusual brick-shaped pore structure. Upon the exploration of the COF as a chemical sensor, it revealed a high selectivity and sensitivity for Cu(II) ions through a PET-based fluorescence quenching. However the presence of trivalent metal ions including Fe(III), Ga(III), Al(III), or Cr(III) increased the fluorescence intensity of the framework (Figure 13B). To verify the enhancement mechanism, zeta potential spectra of CorMeO-COF and metal coordinated COF, M@CorMeO-COF, were compared. The potential of M@CorMeO-COF was substantially higher than CorMeO-COF, which can increase interlayer charge repulsion and impair π−π stacking. The weakening of interlayer contacts was also demonstrated by the blue shift of UV-vis absorption for CorMeO-COF in THF dispersion containing M metal ions. The drop-in diffraction intensities of the PXRD patterns of CorMeO-COF and M@CorMeO-COF also suggested the weakening of the π−π stacking. This led to the conclusion that the enhancement in fluorescence intensity was caused by the reduction ACQ effect, which is typically present in bulk COF materials, leading to an increase in the photoluminescence efficiency following the binding of metal ions in the corrole moieties of the framework. The remarkable fluorescence on/off behavior exhibited by CorMeO-COF makes it an exceptional chemosensor with great promise for metal ion detection applications. This research also opens up new possibilities for employing corrole-based porous materials for the selective detection of significant metal ions.

**FIGURE 14:**
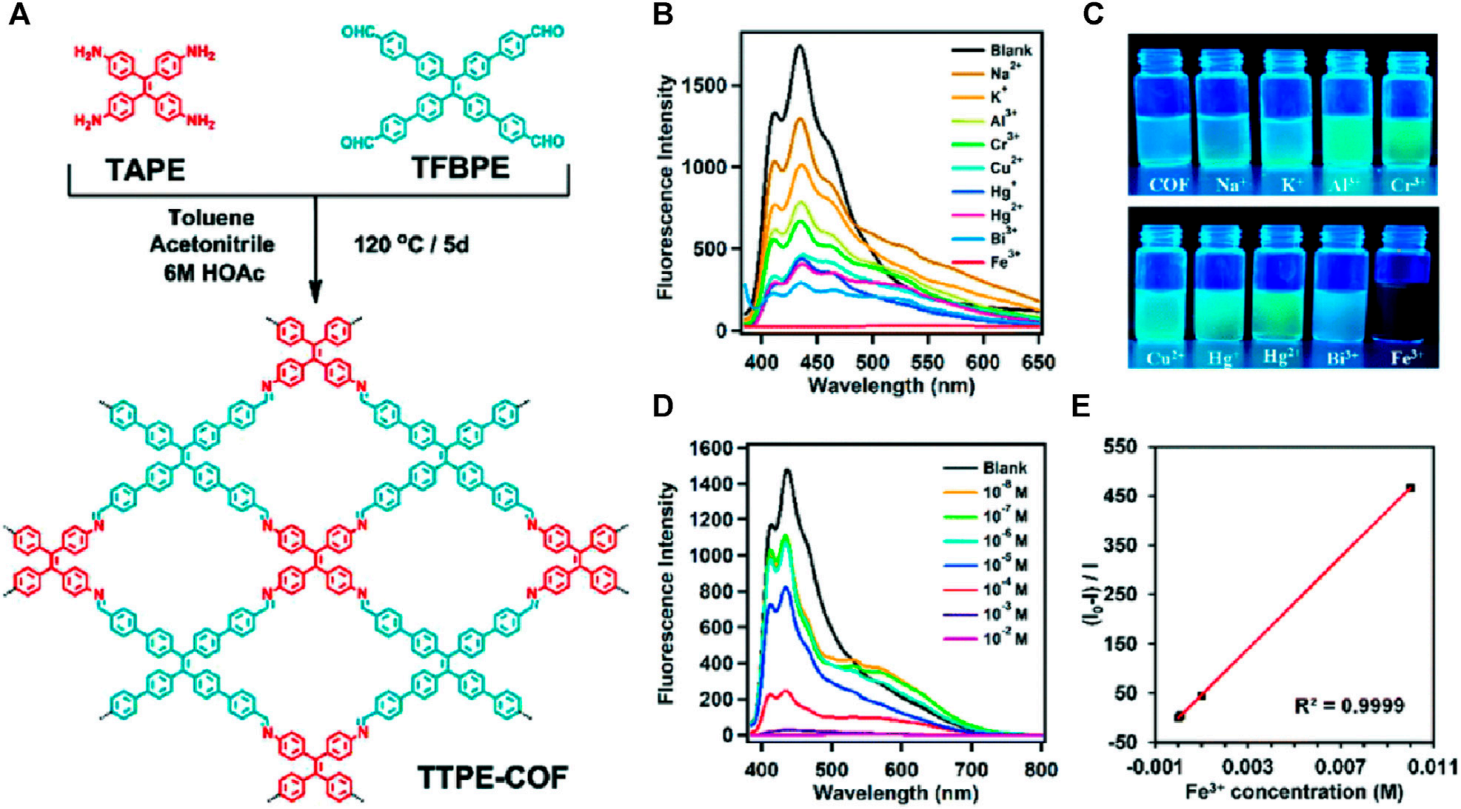
**(A)** The synthetic route of the TTPE-COF; **(B)** Fluorescence emission spectra of the TTPE-COF in the presence of different metal ions; **(C)** the fluorescence photographs under a 365 nm UV lamp; **(D)** the changes in the fluorescence spectrum of the TTPE-COF in the presence of a low concentration of Fe(III); **(E)** Corresponding Stern–Volmer plot. Reprinted with permission from (Cui et al., 2021). Copyright 2021 Royal Society of Chemistry.

A tetraphenylethylene-based covalent organic framework (TTPE-COF) was synthesized through Schiff base reaction between 4′,4″,4″,4‴′-(ethene-1,1,2,2-tetrayl)tetrakis([1,1′-biphenyl]-4-carbaldehyde) (TFBPE) and 1,1,2,2-tetrakis(4-aminophenyl) ethene (TAPE) in anhydrous toluene and acetonitrile using acetic acid as a catalyst (Figure 14A) (Cui et al., 2021). The TTPE-COF’s sharp and intense IR peaks of C=O and -NH_2_ vanished, and a new peak of C=N stretching vibration at 1,602 cm^-1^ indicated that the framework was formed. This indicates that there occurred a condensation reaction between the monomers. Solid-state ^13^C CP/MAS NMR spectroscopy also confirms a distinctive resonance signal of imine carbon at *δ* = 156 ppm, providing additional evidence for the presence of imine bonds (C=N) in the TTPE-COF structure. In addition to being used as an adsorbent for volatile organic contaminants such as benzene and toluene due to its high porosity and structured framework, TTPE-COF also exhibited emission at λ = 585 nm when excited at a wavelength of 530 nm. Further investigations on the fluorescence sensing properties using solutions of various metal ions in water showed that except for Fe(III) ions, other metal ions had little to no impact on the fluorescence emission of TTPE-COF (Figure 14B).

With a quenching efficiency of 99.6% for Fe(III) ions, the changes in fluorescence intensity were easily visible under a 365 nm UV lamp (Figure 14C). After adding various alkali metal ions as well as transition metal ions to the TTPE-COF, only the vial containing Fe(III) ion exhibited no fluorescence emission which makes this a potential visual sensor for Fe(III) ion. The d orbitals of Fe(III) ions make them very efficient electron acceptors and they can accept electrons from the luminescent moiety of TTPE-COF upon light excitation which leads to a donor-acceptor type interaction that results in fluorescence quenching due to energy transfer. The strong binding ability of Fe(III) leading to a special coordination interaction with TTPE-COF ions accounts for the difference in fluorescence quenching compared to other metal ions (Wang et al., 2010; Wang et al., 2018). Fluorescence titration studies showed a gradual decrease in the emission intensity and a linear Stern–Volmer relationship (Figures 14D, E) with a *K*_SV_ value of 46,681 Lmol^−1^ and a LoD value of 3.07 μM revealing the high sensitivity of the framework. In addition to being highly sensitive, this TTPE-COF framework was able to detect Fe(III) ions even in the presence of other metal ions and also showed high regeneration capabilities.

After centrifugation and washing in ethanol, the TTPE-COF sensor was ready to be used again and gave promising quenching efficiencies even after 5 cycles. TTPE-COF can act as a promising candidate as a highly selective, sensitive practically applicable, and cheap visual sensor that can detect Fe(III) ions.

**FIGURE 15:**
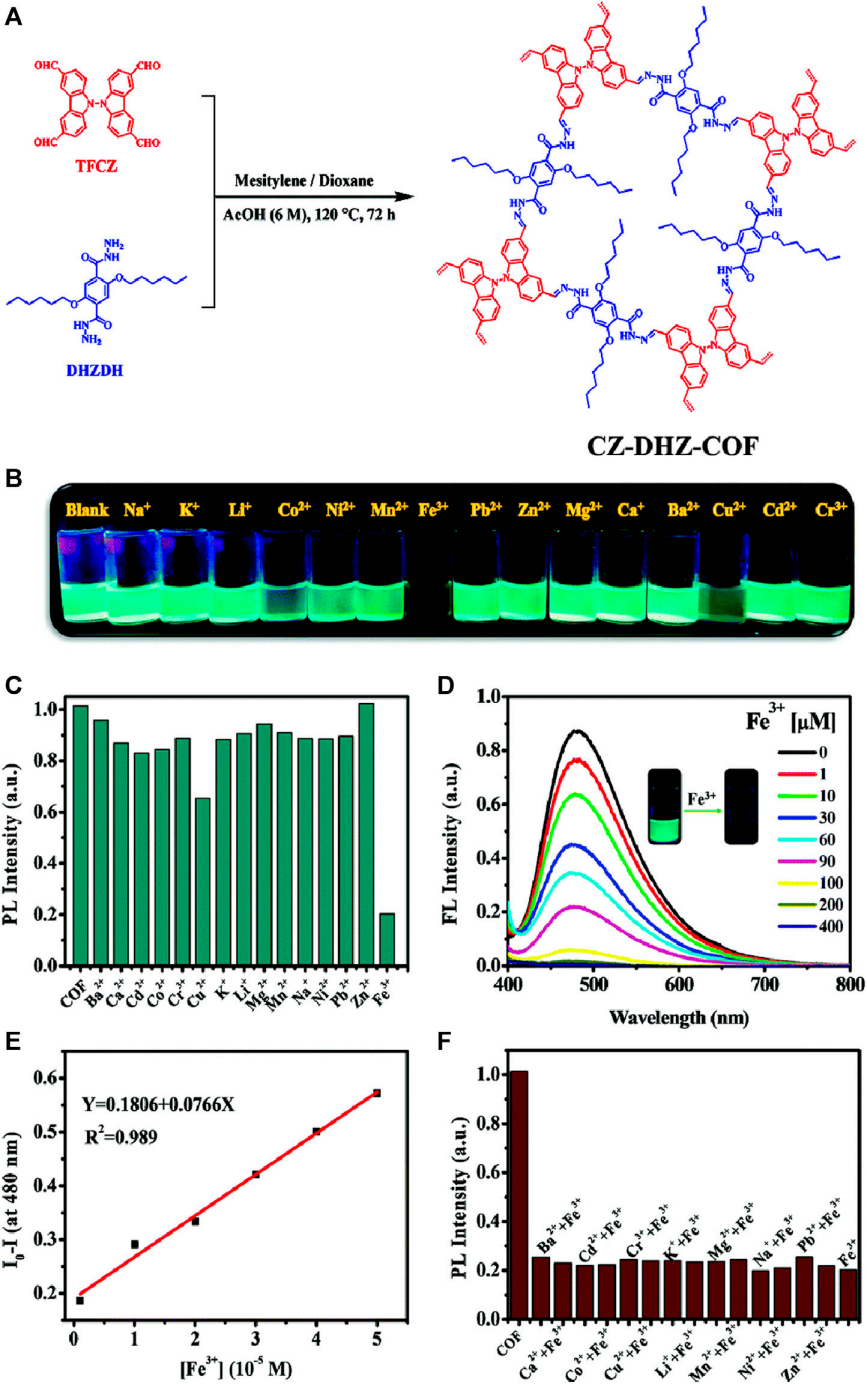
**(A)** The synthetic route of the CZ-DHZ-COF; **(B)** Visual sensing ability of CZ-DHZ-COF towards various metal ions in an ethanol solution; **(C)** Fluorescence emission responses of the CZ-DHZ-COF in the presence of different metal ions; **(D)** Changes in fluorescence emission spectra of the CZ-DHZ-COF dispersed in ethanol containing different concentrations of Fe(III) Inset: The photographs of the CZ-DHZ-COF under UV irradiation at 365 nm before and after titration with Fe(III) ions. **(E)** A linear relationship between the fluorescence intensity of the CZ-DHZ-COF and the concentration of Fe(III). **(F)** Competition experiments: the normalized fluorescence intensity of the CZ-DHZ-COF in the presence of Fe(III) and other metal ions. Reprinted with permission from (Gong et al., 2022). Copyright 2022 Royal Society of Chemistry.

Carbazole derivatives have gained great research interests in recent years due to their rich photophysical properties and potential for various technologically related applications (Mohamed et al., 2019; Xu et al., 2019). The incorporation of carbazole units into COFs containing hydrazone linkages can potentially result in effective fluorescence sensing methods. Based on this logic, Zhang and group prepared a bi-carbazole containing luminescent COF, CZ-DHZ-COF, with hydrazone linkage, which showed emission properties in solution (dispersed in ethanol) and exhibits a bright solid-state fluorescence emission (Figure 15A) (Gong et al., 2022). The as-synthesized CZ-DHZ-COF powder exhibited rapid fluorescent quenching for acid vapor and Fe(III) ions. Since COF powder has limitations to be used as a vapor sensor directly, aerogel which is known to have a highly porous structure with large surface area was used as a substrate for support thus further expanding the practical applications of the COF. Since aerogels are nonfluorescent, it will not affect the fluorescent sensing properties of the COF. CZ-DHZ-COF was combined with a chitosan aerogel CZCA with a designable shape. The advantage of CZCA is that it can be easily molded in various shapes and still retain the fluorescence sensing properties of the COF powder. They performed sensing of Fe(III) in both solution states as well as for the aerogel sample. Upon recording the emission spectra of various metal ions in ethanol solution after dispersing CZ-DHZ-COF, remarkable quenching was observed for Fe(III) ions as the bright cyan emission turned dark very quickly (Figures 15B, C). The efficient Fe(III) ions coordination with the many N, O-chelating sites incorporated into the COF pore wall structure, results in the energy or electron transfer-mediated quenching of fluorescence. The fluorescence intensity of the CZ-DHZ-COF decreased as the Fe(III) concentration increased (Figure 15D) and at a lower concentration range, a linear Stern–Volmer relationship was obtained with a LoD of 3.89 × 10^−7^ M. The fluorescent sensing of the aerogel sample was found to be not as effective since the ethanol suspension as the coordination between Fe(III) and COF was prevented due to the adsorption and coordination between aerogel and Fe(III) ions (Figures 15E, F). The fluorescent change was still clear enough to be observed and this emphasizes the importance of creating suitable composite materials of COFs with high luminescence for practical sensing applications.

**FIGURE 16:**
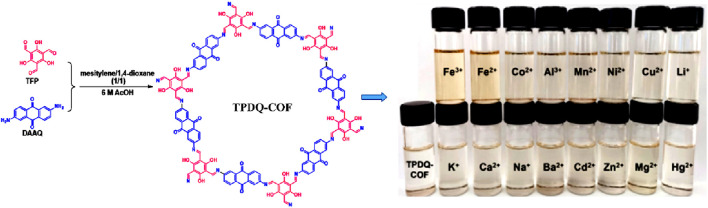
The synthetic route of the TPDQ-COF and its colorimetric sensing responses towards various metal cations. Reprinted with permission from (Xiu et al., 2023). Copyright 2022 Royal Society of Chemistry.

Very recently, the Guang group developed a calorimetric sensor that has a high selectivity for Fe(II) and Fe(III) ions (Xiu et al., 2023). A highly crystalline COF, TPDQ-COF, was synthesized using 1,3,5-Triformylphloroglucinol (TFP) and 2,6-Diaminoanthraquinone (DAAQ) (Figure 16). PXRD measurements confirmed AB stacking and were consistent with the simulated AB stacking model as well as literature reports. The formation of the N=C bond in the TPDQ-COF was realized by the disappearance of N-H peaks at 3,430 and 3,340 cm^−1^ and C=O peaks at 1,640 cm^−1^ in the FT-IR spectra. Further confirmation of the structure was done by XPS and SEM studies. TPDQ-COF demonstrated a colorimetric response for only Fe(II) and Fe(III) ions and upon measuring UV-visible absorbance spectra of the various metal ions, Fe(II) and Fe(III) ions exhibited a remarkable enhancement. TPDQ-COF suspension in DMF exhibited exceptional resistance to interference from other cations as well as anions and a highly selective enhancement response was observed. Being able to detect Fe(II) and Fe(III) ions with very low LoD values {0.691 μM for Fe(II) and 0.714 μM for Fe(III)}, TPDQ-COF could perform as a highly sensitive detection unit which was even able to distinguish between Fe(II) and Fe(III) ions using a simple colorimetric method. The addition of potassium ferricyanide solution to the suspensions led to Fe(II) ion forming a complex with ferricyanide and the yellow-colored solution turned to steel blue while the yellow color of Fe(III)@TPDQ-COF increased. A detailed idea about the mechanism of sensing was revealed when the addition of EDTA to the TPDQ-COF suspension of Fe(II)/Fe(III) ions reversed their UV absorption intensities. This led to the conclusion that the coordination effect followed by a ligand-to-metal charge transfer (LMCT) transition in the complex of TPDQ-COF with Fe(II)/Fe(III) resulted in enhanced UV absorption (La Cognata and Amendola, 2023). This was again verified using XPS studies and the coordination arrangement of TPDQ-COF with Fe(II)/Fe(III) as well as the complex formation were further elucidated by the DFT calculation findings.

## 5 Conclusion and outlook

In summary, COPs and COFs as a category of organic porous polymers, have experienced a resurgence in interest and applications in sensor chemistry. While COPs/COFs have been known for many decades, their potential applications have been realized and explored more extensively in recent times. Their advancements have led to their utilization in addressing environmental issues, serving as adsorbents, filter membranes, chemosensors, and catalysts. Moreover, their potential extends to various other fields, including gas adsorption, energy storage, optoelectronics, and drug delivery. The versatile nature of COPs and COFs paves the way for innovative solutions in diverse scientific and technological endeavors. Metal sensing is one particular area where COPs/COFs have shown great potential. With their ability to selectively interact with metal ions, COPs/COFs offer a pathway to overcome the challenges associated with metal detection. They can be tailored to exhibit high selectivity towards specific metal ions, enabling precise and reliable detection in complex sample matrices. One key attribute that sets COPs/COFs apart is their ability to provide a large number of identical binding sites within a single extended framework, offering exceptional sensing capabilities. By incorporating specific functional groups or ligands into the COPs/COFs structures, it becomes possible to target and detect specific metal ions with minimal interference from other components in the sample. This selectivity ensures accurate and reliable measurements, contributing to the overall advancement of sensing technologies. Furthermore, COPs and COFs offer several advantages over traditional sensing materials. Their tuneable porosity, surface chemistry, and structural diversity allow for tailoring their properties to specific sensing requirements. This review focuses on the diverse applications of COPs and COFs in the realm of fluorescence-based metal sensing, with particular emphasis on the detection of Fe(II) and Fe(III) ions. The ability of COPs/COFs to interact selectively with Fe(II) and Fe(III) ions holds significant implications for a wide range of applications. This includes environmental monitoring, where the detection of iron ions contamination in water sources is crucial for ensuring safe drinking water. In addition, the biomedical field can greatly benefit from COF-based sensors for monitoring iron levels in biological samples, aiding in the diagnosis and treatment of various health conditions. Table 1 summarizes the fluorescence sensing performances of various COPs and COFs-based fluorescence sensors for the selective detection of iron ions. Here we have also explored various experimental techniques and methodologies employed in the design and fabrication of different COF-based sensors. Additionally, the review delves into the underlying mechanisms and interactions between COFs and Fe(III) ions, elucidating the basis for their exceptional sensing capabilities. Furthermore, we highlight the potential challenges with existing COFs-based sensors and develop suitable sensor systems for real-time monitoring of the concentration of iron ions.

**TABLE 1:** Fluorescence sensing properties of luminescence COPs and COFs-based sensors for Fe(III) ions discussed herein.

Sensors	BET surface area (m^2^/g)/Pore size (nm)	Emission maxima (nm)	Sensing medium/Target analyte	LoD	*K*_SV_ (M^–1^)	(Ref.)
COP-100	4.3 to 82.3/27	420	DMF/Fe(II), Fe(III)	2.13 to 2.45 × 10^−7^ M	2.58 × 10^4^ (Fe(II)) 2.97 × 10^4^ (Fe(III))	Özdemir et al. (2015)
POP-HT	414.1/0.41	478	Aqueous/Fe(III)	5 ppm	–	Ma et al. (2016)
TPA-COP	-	498	THF/Fe(III)	4.3 × 10^−7^ M	-	Guo et al. (2018)
P[5]-TPE-CMP	6.99/-	537	Aqueous DMF/Fe(III)	-	-	Li et al. (2018)
UHCOP	-/7.98	510	Aqueous/Fe(III)	2.5 × 10^−6^ M	-	Ji et al. (2022)
LNU-22	524/0.78–1.44 nm (microporous) 2.15–3.85 nm (mesoporous)	480	THF/Fe(III)	2.86 × 10^−5^ M	8.80 × 10^2^	Yan et al. (2022)
LNU-24	71/3.78-4.68	508	THF/Fe(III)	5.34 × 10^−6^ M	2.13 × 10^3^	Yan et al. (2022)
PI-COF-201	3.929/1.34	395	DMF/Fe(III)	0.13 μM	3.23 × 10^3^	Wang et al. (2017)
PI-COF-202	9.161/1.41	462	ACN/Fe(III)	0.22 μM	3.54 × 10^3^	Wang et al. (2017)
Bth-Dma	392/0.44	518	H_2_O/Fe(III)	0.17 μM	2.3 × 10^4^	Chen et al. (2019)
COF-TT	528/0.52	490	Aqueous/Fe(III)	3.69 × 10^−4^ M	1.3 × 10^4^	Li et al. (2019)
TT-COF	646/2.37	425	Ethanol/Fe(III)	8.4 × 10^−5^ M	5.63 × 10^3^	Zhang et al. (2022)
TD-COF	100.62/1.0455	510 and 630	Aqueous/Fe(III)	10.9 × 10^−9^ M	-	Zhang et al. (2023)
CorMeO-COF	634/1.74	655	THF/Cu(II)	1.13 × 10^−6^ M	4.68 × 10^4^	Li et al. (2020)
TTPE-COF	681.27/2.93	585	-/Fe(III)	3.07 × 10^−6^ M	4.66 × 10^4^	Cui et al. (2021)
CZ-DHZ-COF	−/−	488	-/Fe(III)	3.89 × 10^−7^ M	-	Gong et al. (2022)
TPDQ-COF	443/1.007	365	Aqueous/Fe(III) and Fe(II)	6.91 × 10^−7^ M (Fe(II)) and 7.14 × 10^−7^ M (Fe(III))	-	Xiu et al. (2023)

Despite the great research advancements in utilizing COPs and COFs as fluorescence sensors for the detection of various analytes including metal cations, several drawbacks need to be addressed to realize the practical sensing applications. Most of the COPs and COFs-based sensors highlighted in this article are water-insoluble and fluorescence sensing studies were performed in non-aqueous solvent medium. To meet the practical applications, it is advisable to make water-soluble fluorescence sensors. The solubility of COPs and COFs can be improved by introducing ionic or water-solubilizing functional units within the polymeric chains. Any sensor system must be reversible and can be used multiple times for sensing target analytes and at the same time retain the other sensing properties like sensitivity and selectivity for particular analytes. The reversibility is another bottleneck for COPs and COFs. Several of the organic polymer-based sensors lack reversibility and also lose their sensing potency after testing for reusability. Therefore, it is essential to explore the reusability aspect of COPs and COFs for their real-life sensing applications. Another major limitation of COPs and COFs-based sensors is their molecular aggregation in solution which eventually leads to the formation of less-emissive materials. This can be addressed by the installation of a macrocycle as a building unit within the framework structure. Finite macrocycles with non-collapsable backbones attracted great interest as versatile building blocks for the construction of COPs and COFs because of their facile synthesis and aesthetic appearances (Sahoo and Crisponi, 2019; La Cognata and Amendola, 2023). The macrocyclic structure can prevent molecular aggregation and retain the intrinsic photophysical properties of COPs and COFs. In addition, the macrocyclic building units will provide a confined internal cavity that can be exploited for size and shape-selective encapsulation of target analytes thus, improving the selectivity of COPs and COFs. This review identifies various opportunities for further research, such as improving sensor selectivity, enhancing detection limits, and exploring novel architectures for advanced and practical fluorescence-based sensing applications.
